# Genome-wide analysis of R2R3-MYB transcription factors family in the autopolyploid *Saccharum spontaneum*: an exploration of dominance expression and stress response

**DOI:** 10.1186/s12864-021-07689-w

**Published:** 2021-08-18

**Authors:** Yuan Yuan, Xiping Yang, Mengfan Feng, Hongyan Ding, Muhammad Tahir Khan, Jisen Zhang, Muqing Zhang

**Affiliations:** 1 State Key Laboratory for Conservation and Utilization of Agro Bioresources, Guangxi Key Laboratory for Sugarcane Biology, Guangxi University, Nanning, 530005 China; 2 Nuclear Institute of Agriculture (NIA), Tando Jam, 70060 Pakistan; 3 Fujian Agricultural and Forestry University, Fuzhou, 350002 China

**Keywords:** MYB, Sugarcane, Expression analysis of stress, Allelic diversity

## Abstract

**Background:**

Sugarcane (*Saccharum*) is the most critical sugar crop worldwide. As one of the most enriched transcription factor families in plants, MYB genes display a great potential to contribute to sugarcane improvement by trait modification. We have identified the sugarcane MYB gene family at a whole-genome level through systematic evolution analyses and expression profiling. *R2R3-MYB* is a large subfamily involved in many plant-specific processes.

**Results:**

A total of 202 *R2R3-MYB* genes (356 alleles) were identified in the polyploid *Saccharum spontaneum* genomic sequence and classified into 15 subgroups by phylogenetic analysis. The sugarcane MYB family had more members by a comparative analysis in sorghum and significant advantages among most plants, especially grasses. Collinearity analysis revealed that 70% of the *SsR2R3-MYB* genes had experienced duplication events, logically suggesting the contributors to the MYB gene family expansion. Functional characterization was performed to identify 56 *SsR2R3-MYB* genes involved in various plant bioprocesses with expression profiling analysis on 60 RNA-seq databases. We identified 22 MYB genes specifically expressed in the stem, of which *RT-qPCR validated MYB43*, *MYB53*, *MYB65*, *MYB78*, and *MYB99*. Allelic expression dominance analysis implied the differential expression of alleles might be responsible for the high expression of MYB in the stem. *MYB169*, *MYB181*, *MYB192* were identified as candidate C_4_ photosynthetic regulators by C_4_ expression pattern and robust circadian oscillations. Furthermore, stress expression analysis showed that *MYB*36, *MYB*48, *MYB*54, *MYB*61 actively responded to drought treatment; 19 and 10 MYB genes were involved in response to the sugarcane pokkah boeng and mosaic disease, respectively.

**Conclusions:**

This is the first report on genome-wide analysis of the MYB gene family in sugarcane. SsMYBs probably played an essential role in stem development and the adaptation of various stress conditions. The results will provide detailed insights and rich resources to understand the functional diversity of MYB transcription factors and facilitate the breeding of essential traits in sugarcane.

**Supplementary Information:**

The online version contains supplementary material available at 10.1186/s12864-021-07689-w.

## Background

Modern cultivated sugarcane (*Saccharum spp.*) is the primary source of sugar for the world. It is the topmost crop concerning total biomass production and is listed among the 10 most valuable crops [1]. Sugarcane, having a complex genetic background resulting from polyploid interspecific hybrids, was first domesticated approximately 10,000 years ago in New Guinea. *Saccharum spontaneum* contributes to 10–15% chromosomes in modern sugarcane cultivars, endowing the characteristics such as disease resistance and ratooning capacity [2]. The genome of haploid *S. spontaneum* has been assembled to the chromosome level and used as the reference genome of sugarcane [3]. Because of the development of multiple transcriptome models in recent times, including those for different tissues, developmental stages, and under various stress treatments, massive RNA-seq data are available, which can provide detailed insights and rich resources for studying sugarcane genes functions.

Transcription factors recognize specific DNA motifs in upstream regions of the genes to regulate their expression. MYB genes constitute one of the most prominent families of plant transcription factors and characteristically possess highly conserved Myb DNA-binding domains, forming a helix-turn-helix structure of about 52 amino acids [4]. MYB genes can be divided into four categories, including MYB-related, R2R3-MYB, R1R2R3-MYB, and atypical MYB, depending on the number of adjacent MYB repeats (R). Proteins with a single or a partial MYB repeat, generally located at either ends or middle of the peptide chain, are MYB-related.

MYB-related proteins include critical telomere binding proteins in maintaining the integrity of the chromosome structure [5]. Moreover, they also play an essential role in regulating gene transcription, e.g., the GARP family of plant Myb-related DNA binding motifs is involved in organ polarity in Arabidopsis [6]. Further, *CIRCADIAN CLOCK ASSOCIATED*1 (*CCA*1) and *LATE ELONGATED HYPOCOTYL* (*LHY*) genes regulate the plant circadian clock [7]. A small number of members of R1R2R3-MYB genes are found in higher plants. Interestingly, plant R1R2R3-MYB genes share a similar function of regulating the cell cycle control with the animals [8]. R1R2R3-MYB has also been involved in cell differentiation [9] and plant stress tolerance [10].

**Fig. 1:**
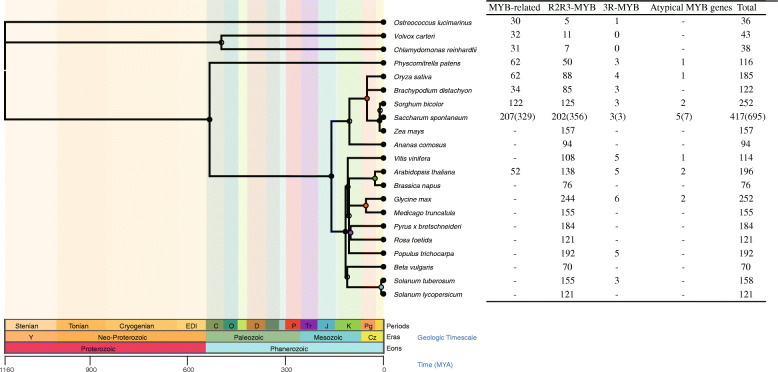
Phylogenetic tree of diverse species showing the number of MYB family. The phylogenetic tree reflects the evolutionary relationship and divergence time of various species in plants through the TimeTree database (http://www.timetree.org). Linear scale Time MYA (millions of years ago) and Geologic Timescale were shown at the tree’s bottom. These species contain Green algae, Grasses (red node), Cruciferous (green node), Leguminosae (orange node), Rosaceae (purple node), Chenopodiaceae (blue node), and others altogether 11 lineages. MYB gene family could be divided into four subfamilies according to the number of Myb domains. The available information of the MYB gene family was obtained from the reported literature. The short line represented undetermined. The MYB families were estimated by performing profile searches using a combination of the HMMER3 program (PF00249) and NCBI-CDD database for *S. spontaneum* and *S. bicolor*. *Myb_DNA-binding* domain (PF00249) was downloaded from Pfam (Finn et al., 2010). *S. spontaneum* MYB gene in brackets was identified throughout the genome and contained alleles. A single set of genes were shown in outsides for each subfamily. Different alleles of one gene might be divided into different subfamilies. Thus, to better classification, a single set of genes did not distinguish this condition that one gene was in different subfamilies. Geologic Periods: C(Cambrian), O(Ordovician), D(Devonian), P(Permian), Tr(Triassic), J(Jurassic), K(Cretaceous), Pg(Paleogene)

Atypical MYB proteins contain four or more adjacent MYB repeats (R). These proteins have been found to encode in a few plants, e.g., *Arabidopsis thaliana, Oryza sativa*, *Vitis vinifera*, *Glycine max*, *Physcomitrella patens* (data sources displayed in Materials and Methods 2.1), as shown in Fig. 1. Only a few reports have been published about atypical MYB proteins by now, and the role of these proteins in the plant bioprocesses is mainly unknown. MYB transcription factors binding specific DNA sequence (CAACG/TG) result from domain structure that is formed by two closely packed amino acid sequence repeats(R) [11]. When the MYB gene contains at least two MYB repeats (R), it has transcription factor characteristics and explicitly recognizes the DNA motifs to regulate the gene transcription. R2R3-MYB proteins are the largest subfamily of MYB transcription factors in plants, as well as in *S. spontaneum* (Fig. 1). R2R3-MYB is characterized by two MYB repeats and the presence of a single amino acid (Leu) in the first (R2) repeat [12]. R2R3-MYB has two MYB repeats and a single amino acid (Leu) inserted in the first (R2) repeat. The R2R3-MYB family’s expansion originated from the R1R2R3-MYB gene ancestor when losing the R1 repeat sequences during evolution [13] and benefiting from gene duplication events [14].

MYB genes are widely involved in plant-specific processes, such as differentiation [15], hormone response [16], secondary metabolism [17], environmental stress tolerance [18], and diseases resistance [19, 20]. At least four MYB genes are involved in lignin biosynthesis in *Arabidopsis* by activating key regulator genes related to secondary cell wall formation [21]. Under environmental stress, MYB genes have been reported to function in response to adverse stress in *Arabidopsis*. Moreover, *AtMYB2* and *AtMYB96* function as transcriptional activators in ABA-inducible gene expression under drought stress [22]. *AtMYB96* mediates abscisic acid signaling, induces pathogen resistance response by promoting salicylic acid biosynthesis, and provides drought tolerance via controlling the cuticular wax biosynthesis [20, 23].

This study focused on the *R2R3-MYB* gene family in the *S. spontaneum* published sugarcane genome. We provided a detailed overview of phylogenetic relationship, gene structure, regulatory elements, expression profiles, allelic evolution, and functional characterization based on abundant transcriptome data. Our study systematically explored the evolutionary dynamics and functional diversification of *SsR2R3-MYB* genes and could facilitate future research on sugarcane MYB transcription factors.

## Results

### Genome-wide identification of *R2R3-MYB* genes and classification in *S. spontaneum* genome

Based on the functional annotation of the *Myb_DNA-binding* domain (PF00249), a total of 418 MYB genes (695 alleles) were identified in *the S. spontaneum* genome by combining the HMMER program and NCBI-CDD database (Fig. 1). The SsMYB gene family was classified into four distinct subfamilies, including 207 MYB-related (329 alleles), 202 R2R3-MYB (356 alleles), 3 R1R2R3-MYB (3 alleles), and 5 Atypical MYB (7 alleles) genes (detailed data presented in supplementary Table S1). Total 122 SbMYB-related, 125 SbR2R3-MYB, three SbR1R2R3-MYB, and two atypical SbMYB genes were also identified to increase the understanding of the *SsR2R3-MYB* genes in sorghum (Table S3).

To analyze the plant MYB genes thoroughly, 20 species representing 11 lineages were screened to construct a plant phylogenetic tree with *S. spontaneum*, including Green algae, Bryophyta, Gramineae, Cruciferous, Leguminous, Rosaceae, Solanaceae, and others. The tree topology reflected the phylogenetic relationship of these species and divergence time (Fig. 1). Plant phylogeny showed that the higher plants possessed more MYB genes than the lower plants, such as green algae (e.g., *Ostreococcus lucimarinus, Volvox carteri,* and *Chlamydomonas reinhardtii*). A significant expansion of MYB genes was observed after the Cambrian (about 540 ~ 480MYA), demonstrating an explosive biological diversification episode near the early period [24]. Most of the phylogenetic nodes of plant species were observed in the Cretaceous, a geological period when a typical global warming climate contributed to the terrestrial species diversity [25]. Compared with the other four kinds of grasses, *S. spontaneum* had one of the most significant MYB genes as predicted by PlantTFDB. One reason is the tetraploid nature of the autopolyploid *S. spontaneum* (octoploid). However, when corrected for ploidy level, the number of *SsR2R3-MYB* genes in *S. spontaneum* was still higher than most of the species, including *Arabidopsis thaliana* and other grass species. The number of MYB genes with a phylogenetic tree of the plant species indicated the expansion of MYB genes from alga to land plants, consistent with previous reports [26].

**Fig. 2:**
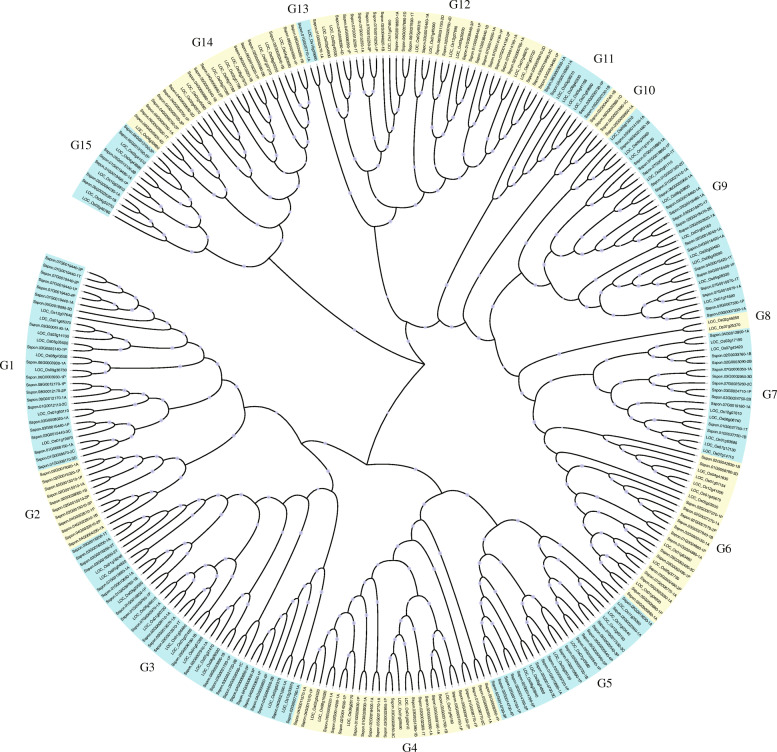
Phylogenetic relationships of *R2R3-MYB* subgroup members from *S. spontaneum* and rice. A phylogenetic tree of *R2R3-MYB* proteins from *S. spontaneum*, rice, and Arabidopsis was constructed using FastTree2 with the maximum likelihood method. The sugarcane *R2R3-MYB* families were clustered into 15 subgroups, named G1 to G15. The subgroups were covered with colorful backgrounds. Red dots represented SsMYBs, and adjacent genes on a branch were tagged one dot

Maximum likelihood phylogenetic tree of *R2R3-MYB* genes from *O. sativa* and *S. spontaneum* showed that the sugarcane genome contained 15 subgroups (G1-G15) *OsR2R3-MYB* genes (Fig. 2, Table S2) with each distributing rice MYBs. Sugarcane and rice diverged in the *Paleogene* (67-26MYA) (Fig. 1); the short divergence time indicated relative conservatism of the ortholog genes. As expected, two species of *R2R3-MYB* genes were evenly distributed in the tree, and most genes in rice clustered with sugarcane, except for G8. However, the number of genes in each clade varied greatly; for instance, the biggest group, G3, contained 38 genes while the group G8 and G13 comprised just two genes. Twenty *SsMYB* genes from three unique subgroups, G2, and G10, did not contain rice genes, indicating the species’ genetic divergence. Besides, the clusters depicted that the sugarcane MYB family exhibited a more significant number of genes than that in rice, showing a significant expansion of the SsMYB family. In addition, we also constructed an ML phylogenetic tree with Arabidopsis (Figure S1). Comparative analysis of two trees showed some differences because of different species, but overall high similarity implied the topology of reliability.

### Analysis of genomic location, gene structure, and regulatory elements

**Fig. 3:**
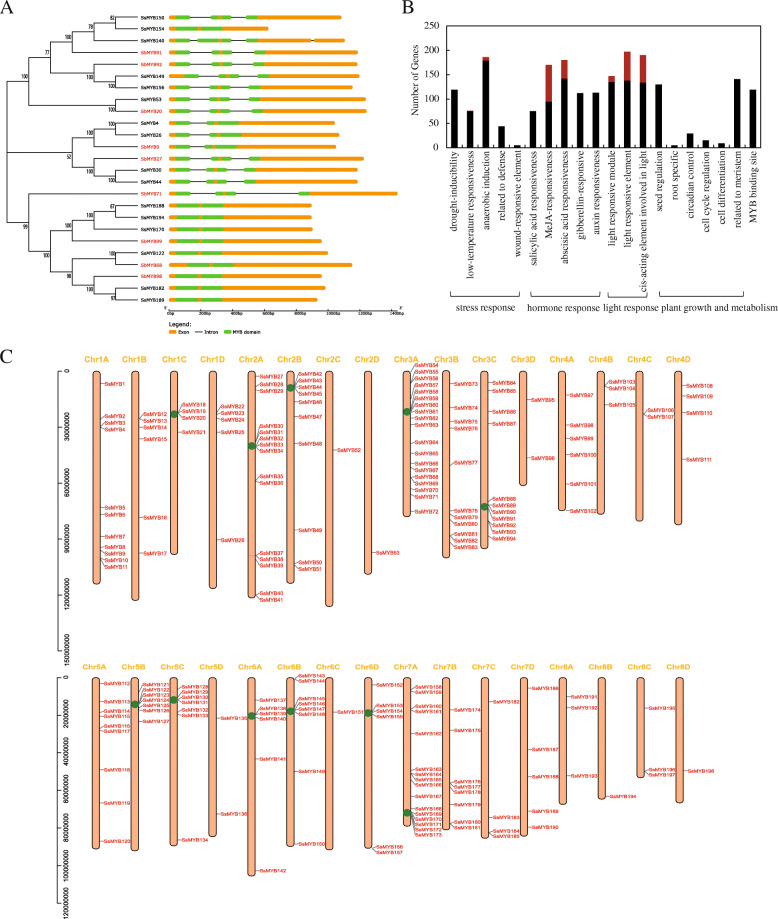
Structure, distribution, and regulatory elements of *SsR2R3-MYB* genes. **a** Comparison of gene structure between *S. spontaneum* and *S. bicolor* based on the phylogenetic tree. ClustalX performed the sequence alignment of *SsR2R3-MYB* and *SbR2R3-MYB* proteins, and the Phylogenetic tree was constructed using MEGA 7.0 with Neighbor-Joining (NJ) method, 1000 bootstrap replicates, Pairwise deletion, and Bootstrap values on the nodes. *SsMYB* gene names are marked black, and *SbMYB* gene names are marked red. Gene sequences were modified to start at the transcription initiation site (ATG), and gene structures were displayed using GSDS2.0 (http://gsds.cbi.pku.edu.cn/). The CDS sequence and intron were represented as fine lines and yellow cylinders, and green cylinders highlighted the MYB domain. The first subgroup was presented here when the estimated phylogenetic relationship of *S. spontaneum* and *S. bicolor* and others were shown in Figure S2. **b** Cis-regulatory elements of *SsR2R3-MYB* gene promoters with diversified plant biological functions. The functions of the predicted cis-regulatory elements covered four main categories: stress response, hormone response, light response, plant growth, and metabolism. The x-axis showed divers plant biological functions, and the y-axis indicated the number of a specific category of genes in that main category. The red rectangle represented the genes containing more than six elements involved in regulating a particular plant function. **c** Distribution of *SsR2R3-MYB* gene members in *S. spontaneum* genome. 202 *SsR2R3-MYB* genes were named according to their physical position on the chromosome and tagged in red font. Yellow font indicated chromosome name, and chromosome was represented as hollow cylinders with length scale (bp) on the left. The green spots displayed a gene enrichment cluster

A total of 202 *SsR2R3-MYB* genes were named in turn according to their physical position on the chromosomes. MYB genes were distributed throughout all 32 chromosomes (Fig. 3c); the autopolyploid *S. spontaneum* genome comprised of eight homologous groups of four members each [3]. The chromosome distribution map showed that the location of the *MYB* genes was not evenly distributed. Most of the *SsMYB* genes were located on Chr3A and Chr7A, encompassing 19 and 16 genes, respectively. About 11 enrichment clusters, tiny fragments on genomic regions containing three MYB genes, were detected, and half of these genes had MYB-binding sites (MBS) depicting potential interaction among each cluster. However, some chromosomes only contained a few MYB genes. For instance, five chromosomes, including Chr2C, Chr2D, Chr6C, Chr8B, and Chr8D, had only one MYB gene.

*S. bicolor* is one of the closest lineages of sugarcane, possessing relatively perfect genomic sequence data [27, 28]. Total 125 *SbR2R3-MYB* genes were identified from the available sorghum genome using a similar method (Fig. 1, Table S3). The diversity of the gene structure might be a shred of evidence regarding the evolution of gene families. The Neighbor-Joining method performed the phylogenetical gene structure analysis using diverse gene information (Fig. 3a and Figure S2). The distribution of the tree branches was consistent with the structural features of the genes. Various sorghum genes were clustered with highly similar *SsR2R3-MYB* genes, e.g., *SbMYB92* clustered with *SsMYB149* and *SsMYB156* while *SbMYB27* was clustered with *SsMYB30* and *SsMYB44*. These results sharpened our understanding of the evolution of gene events during sugarcane polyploidization. A total of 19 *SsR2R3-MYB* genes did not show the presence of intron, including *SsMYB154, SsMYB188, SsMYB194, SsMYB170*, *SsMYB122*, *SsMYB182*, and *SsMYB189*. Many MYB genes demonstrated a domain with a cross-intron structure.

*Cis*-elements in promoter regions play an essential role in controlling transcription and expression, which can deepen the understanding of the regulatory function of MYB genes. Total 2000 bp upstream of transcription initiation site (ATG) was regarded as MYB gene promoters and submitted to the PlantCARE for predicting the motifs. Various motifs from 202 *SsR2R3-MYB* gene promoters were involved in various plant bioprocesses (Fig. 3b). These diversified *cis*-regulatory elements could be divided into four main categories in terms of function: stress response, hormone response, light response, and plant growth and metabolism. A high percentage of MYB genes in the anaerobic induction (92%) and drought elements (58.9%) indicated that the MYB genes were more likely to function under these stresses. Moreover, a notable gene, *MYB88,* was found to have 10 LTR motifs, which is a *cis*-acting element involved in low-temperature responsiveness. The significantly enriched LTR elements (5′-CCG AAA-3′) suggested that the *MYB88* gene might be involved in plant metabolic response to cold stress. Many of the MYB genes regulate the plant hormone response, especially methyl jasmonate (MeJA) and abscisic acid (ABA) responsiveness. A total of 75 gene promoters had enriched regulatory elements TGACG-motif (5′-TGACG-3′) and CGTCA-motif (5′-CGTCA-3′) involved in MeJA-responsiveness, while 38 gene promoters enriched regulatory elements ABRE involved in abscisic acid responsiveness. These MYB genes were predicted to regulate MeJA and ABA signaling in plants and function in plant defense and leaf abscission. Furthermore, more than 30 light response-related elements were predicted; for instance, conservative light element G-box was widely present in the upstream sequence of genes. Several regulatory elements were also associated with other plant growth and development functions and regulation of seed growth and meristem development. Genes involved in seed-specific regulation contained the same RY-element (5′-CATGCATG-3′), and the elements involved in meristem expression demonstrated CAT-box (5′-GCC ACT-3′) and NON-box (5′-AGATCGACG-3′) in promoter regions. Finally, 119 genes were scattered on MYB binding sites, and 49 genes showed more than one binding site, suggesting that these genes probably interacted with other MYB genes. Four MYB binding elements were found in 202 *SsR2R3-MYB* promoters, including CCAAT-box (5′-CAACGG-3′), MBS (5′-CAACTG-3′), MBSI (5′-aaaAaaC(G/C)GTTA-3′), and MRE (5′-AACCTAA-3′). There was only one base difference between the former two elements, which accounted for 80% of the total MYB binding elements, suggesting the conservative nature of the sequence CAACG/TG of the MYB binding site. The autoregulation of plant transcription factors is common in one family, which showed sequence-specific interactions of the family [29, 30]. Dof1 binds the *PEPC1* promoter, but *Dof2* blocks the transactivation of *Dof1* [31]. Some identified MYB genes were co-expressed on the STRING network (http://string-db.org/), such as *MYB114*, *MYB 168*, and *MYB167*; *MYB109*, *MYB108*, and *MYB47*. These MYB genes with MYB binding site indicated the potential interaction effects. Co-expression analysis further supports the hypothesis.

### Pervasive gene duplications

**Fig. 4:**
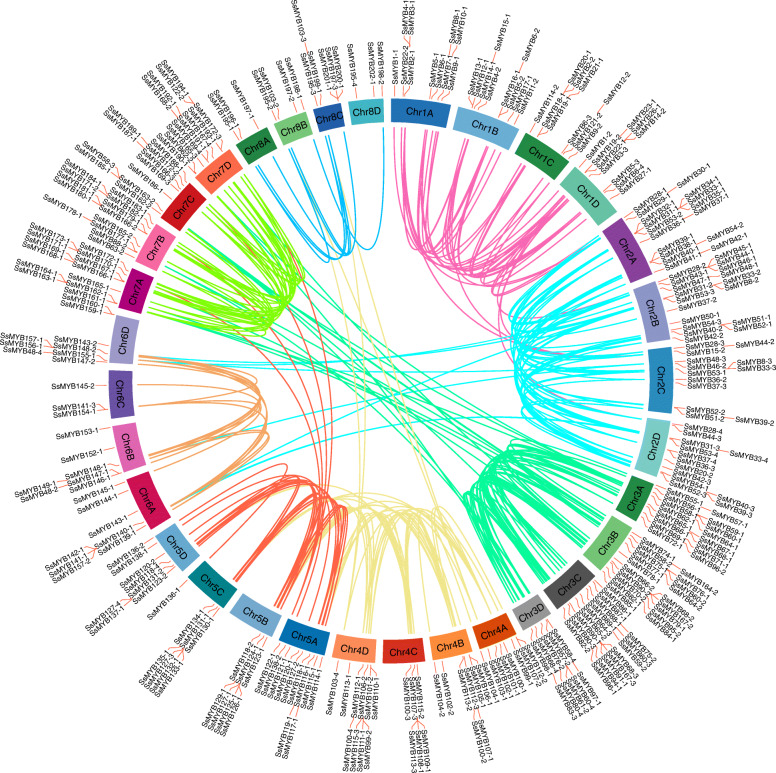
Collinearity relationships of *SsR2R3-MYB* genes on the *S. spontaneum* genome. *SsR2R3-MYB* collinear gene pairs were mapped to their respective locus in the *S. spontaneum* genome in a circular diagram. Genes located on the same chromosome (e.g., Chr1, Chr2, Chr3, Chr4, Chr5, Chr6, Chr7, Chr8) shared one line, and the inter-chromosomal collinear genes pairs were linked with the former linear color

Duplication is a striking feature of the plant genome. Gene duplication in the *R2R3-MYB* gene family occurred during earlier evolution in land plants and contributed to its amplification [32]. We estimated gene duplication events in the *S. spontaneum* genome by collinearity analysis. A total of 274 collinearity pairs of *SsR2R3-MYB* genes were identified by Blastp for all protein sequences and evaluated with MCScanX, including 144 allelic pairs and 130 non-allelic pairs (Fig. 4, Table S4). The collinearity relationships revealed that over half of the collinearity genes were concentrated in Chr 3 and Chr 7. The duplication events for MYB genes were predicted. Total 91 (25.84%) genes were tandem repeats, of which one-quarter of genes were located on Chr 7. Furthermore, 146 (39.88%) genes were identified to derive from segmental duplication events; 28.1% genes on Chr 2 and 33.5% on Chr 3 evolved from segmental duplication (Fig. 4, Table S5). Segmental duplication played a critical role in the MYB gene evolution of *S. spontaneum*, similar to other species. About 66.5% of the *R2R3-MYB* genes derived from gene duplication events drove the MYB gene family expansion.

### Temporal and spatial expression of the *R2R3-MYB* gene family

**Fig. 5:**
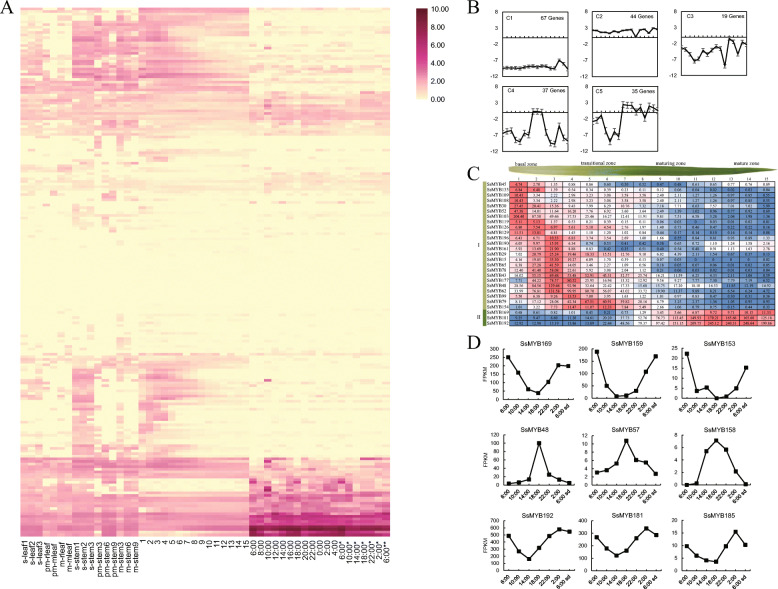
Temporal and spatial expression dynamics of *SsR2R3-MYB* genes. **a** A heatmap showed the expression profile of *SsR2R3-MYB* genes. Columns showed 202 *SsR2R3-MYB* genes, and rows showed developmental stages and tissues, leaf developmental gradient, and circadian rhythm. Abbreviations: s, seeding stage; pm, pre-mature stage; m, mature stage; r leaf, roll leaf; m leaf, mature leaf. Leaf developmental gradient showed 1–15 segment in one leaf blade from base to tip. Circadian rhythm showed 19-time point including first day 2 hours period and second day 4 hours period. Asterisk was used to distinguish the same time point on different days. **b** K-means clustering showing the expression profile of the developmental stages and tissue transcriptome. Five clusters were identified as C1-C5, error bars showing standard deviation. **c** Heatmap of gene-expression levels for co-expression modules along leaf developmental. Each gene (row) in the two co-expression modules (I, II) was sorted according to the point of leaf segment point (column) at which peak expression occurred. **d** The expression of nine MYB genes showed a circadian cycle, and the x-axis indicates different time points on the second day

To characterize the expression profile of MYB transcription factors, the temporally and spatially expression profiles of 202 *SsR2R3-MYB* genes were analyzed using a total of 50 RNA-seq data among three transcriptome models, including tissue and developmental stages, leaf developmental gradient, and circadian rhythm. The expression heatmap showed that most of the MYB genes had low expression levels, but 71% of gene expression values were greater than 1 (FPKM) in at least one RNA-seq sample (Fig. 5a, Table S6). Expression values of 15 *MYB* groups were presented in Table S6, and group G6 genes seemed to be expressed greater than that in the other groups.

Five different expression patterns, i.e., C1-C5, were investigated on the tissue and developmental stages transcriptome by K-means (Fig. 5b). A total of 85 *SsR2R3-MYB* genes belonging to the C1 and C3 clusters had low expression value, particularly C1 genes with almost no expression. On the contrary, C2 cluster genes displayed a relatively higher expression level in all developmental periods of leaf and stem. Interestingly, 37 genes of the C4 cluster were highly expressed in the stem during the seedling stage, the early stage of the stem formation (Figure S3A). Moreover, in the C5 cluster, 35 genes were highly expressed in the stem during each period, probably playing a regulatory role in the stem development (Figure S3B). The clusters indicated that the global gene expression levels in the stem were significantly higher than those in the leaves, suggesting *SsR2R3-MYB* genes might play an essential role in stem tissue. The relative expression of *SsMYB43*, *SsMYB52*, *SsMYB65*, *SsMYB78*, and *SsMYB99* were quantified by RT-qPCR, verifying the results of RNA-seq data (Figure S4A); additionally, *SsMYB3, SsMYB15, and SsMYB157* predominant expressed in the early stage of stem formation depicted as prophase of the stem (Pro-stem), which was much higher than those in other stem nodes and leaf tissues (Figure S4B).

Sugarcane is a typical C_4_ plant with high light use efficiency. The developmental gradient model of grass leaves could be used to study C_4_ photosynthesis and its regulatory factors [33, 34]. The regulatory role of *SsMYB* genes on C_4_ photosynthesis was investigated on the developmental dynamical transcriptome of sugarcane leaf. As suggested by the C_4_ photosynthetic development model, leaves are gradually differentiated for active photosynthesis [33]. A total of 27 differentially expressed *SsR2R3-MYB* genes were detected by the leaf developmental gradient. Most of the genes (Class I) showed an expression profile, illustrating high value in the early stage of leaf development (Figure S5). Only three genes *SsMYB169*, *SsMYB181*, and *SsMYB192* in Class II (Fig. 5c, Figure S5), were identified as putative C_4_-related transcription factors using the method that associated the co-expression pattern with the photosynthetic activity [35]. The expression increased with the development of C_4_ photosynthesis and displayed the highest accumulation at the leaf mature zone. *SsMYB181* and *SsMYB192* shared one haplotype gene *Sspon.07G0015250* with *SsMYB169*, as the tandem genes *SsMYB181* and *SsMYB192* derived from a gene duplication event. Circadian rhythm is another module to study photosynthesis, in which previously identified C_4_-related regulators could also be verified. Nine *SsR2R3-MYB* genes showed a significant association of expression profile with the light-dark cycle (Fig. 5d). Their expression patterns were divided into three modules, each module containing three genes. The expression level of *SsMYB169*, *SsMYB159*, and *SsMYB153* tailed off during the daytime until around 6:00 pm and then gradually recovered till the next cycle. However, *SsMYB48*, *SsMYB57*, and *SsMYB158* raised their expressions during the day and fell at night. Unexpected but reasonable, the preliminary identification of three C_4_-related regulators, *SsMYB169*, *SsMYB181*, and *SsMYB192*, also showed daylight expression pattern, hinting at their involvement in the regulation of circadian rhythm, indicating that the three candidate MYB transcription factors were associated with C_4_ photosynthesis.

### MYB genes involved in response to drought and disease-induced stress

**Fig. 6:**
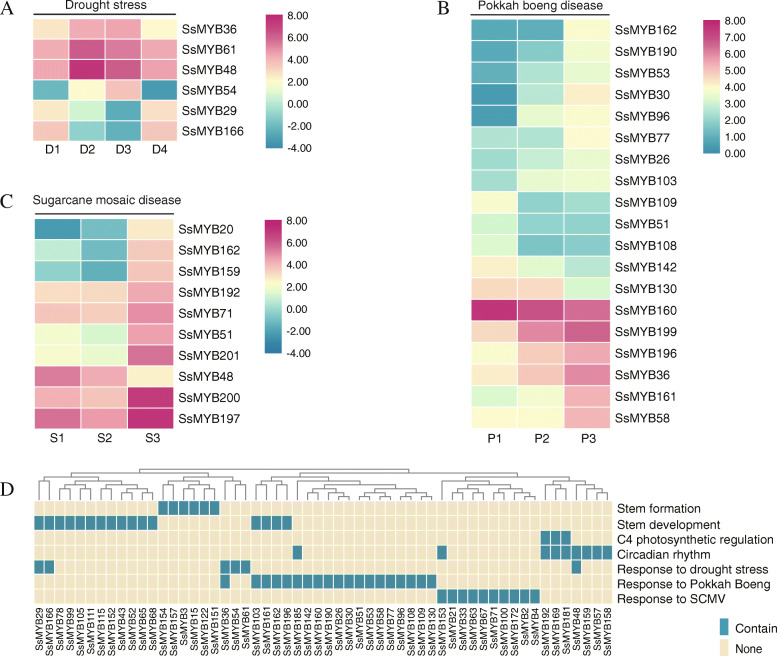
Heatmaps of differentially expressed MYB genes in response to stress conditions and presumed function. The heatmaps of DEGs expression value were shown in (**a**, **b**. **c**) based on RNA-seq data from drought stress, pokkah boeng disease, and sugarcane mosaic disease. DEGs were identified due to their expression having significant variation after suffering stress stimulation (FPKM > 2, fold change > 2, *p*-value < 0.05). Abbreviation: D1, CK; D2, mild; D3, severe; D4, rehydrate. P1, CK; P2, inchoate; P3, advanced. S1, CK; S2, CK detoxify; S3, post-infection. **d** 56 *SsR2R3-MYB* genes with specific expression patterns with putative functionalities. Blue boxes represent this function and yellow with none

The expression patterns of *SsR2R3-MYB* genes were evaluated under environmental stress (biotic and abiotic stress). Six *SsR2R3-MYB* genes with significantly differential expression were responsive to drought induction (Fig. 6a, Table S7). The transcripts of four genes, *SsMYB54*, *SsMYB36*, *SsMYB61*, and *SsMYB48*, rapidly accumulated after drought treatment, but their expression reduced normal level after rehydration. On the other hand, *SsMYB29* and *SsMYB166* showed the opposite trend. Further, the upstream regulatory elements of these six genes contained the MBS element (5′-CAACTG-3′), which was identified as MYB binding site involved in drought-inducibility. Half of these genes retained more than one MBS.

Pokkah boeng disease of sugarcane (PBD) is one of the most severe and devastating diseases caused by the *Fusarium* species complex, a fungal pathogen [36, 37]. Nineteen different MYBs were associated with sugarcane PBD-infection and response (Fig. 6b, Table S7). According to the gene expression profiles, these genes included 14 genes with increased expression in defense response and five genes with reduced expression.

Sugarcane mosaic disease is a highly transmissible viral disease present in cane-growing regions worldwide. Sugarcane mosaic virus (SCMV), belonging to the positive-sense single-stranded RNA viruses, reduces yields by damaging chloroplast and blocking photosynthesis [38, 39]. After SCMV infection, 10 *SsR2R3-MYB* gene expression increased, and one gene, *MYB176*, decreased, suggesting that these MYB genes were involved in defense against SCMV infection (Fig. 6c, Table S7). We discovered that these MYB genes were unique to sugarcane diseases, indicating the defense specificity of MYB genes for conferring the resistance of sugarcane pokkah boeng and mosaic disease.

### Functional characterization

The potential function of *SsR2R3-MYB* genes was predicted on the identified genes with significantly specific expression. Fifty-six *SsMYB* genes were involved in seven plant bioprocesses (Fig. 6d). Six *MYB* genes only expressed during seeding stem and were possibly involved in stem differentiation and formation (Fig. 6a). Three *MYB* genes were identified as candidate C_4_ photosynthesis regulators, and nine genes responded in the circadian clock. Under diverse stresses, it was seen that 6, 19, and 10 *SsR2R3-MYB* genes responded to drought, pokkah boeng disease, and mosaic disease, respectively. Notably, *SsMYB51* and *SsMYB162* illustrated different expression changes between two sugarcane diseases (pokkah boeng and mosaic disease). *SsMYB162* significantly accumulated, actively responding to the infection of two diseases (Table S7). However, *SsMYB51* showed a different expression pattern, negatively responding to pokkah boeng but positively answering SCMV. Moreover, 13 *MYB* genes had more than one putative function, indicating their role in diverse plant bioprocesses (Fig. 6d).

### Allelic expression dominance drove SsMYB to function in stem

**Fig. 7:**
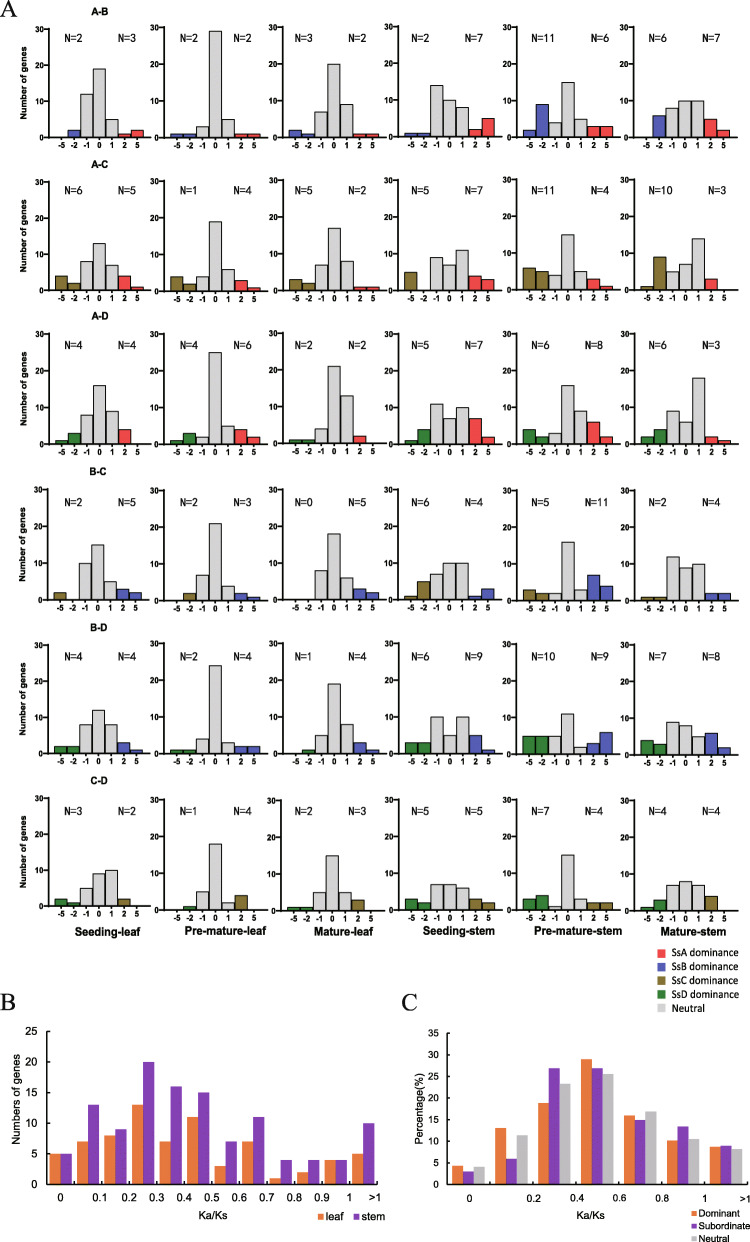
Allelic expression dominance and selective pressure analysis in *SsR2R3-MYB* family. **a** Expression histograms of *SsR2R3-MYB* allelic genes among the tissue and development stage of *S. spontaneum*. N values indicate the number of dominant genes in allelic genes identified *R2R3-MYB* genes. **b** The distribution of Ka/Ks of expressedly dominant genes in leaf and stem. Each KaKs interval corresponds to the number of genes. Ka/Ks median values of leaf and stem were 0.455 and 0.482, respectively. **c** The distribution of Ka/Ks values among allelic genes as dominant, subordinate, and neutral (equal expression level in allelic gene pair). Each KaKs interval corresponds to the percentage of genes. Ka/Ks median values of dominant, subordinate, and neutral genes were 0.491, 0.506, and 0.485, respectively

The transcriptional levels of *R2R3-MYB* allelic genes were compared among different tissues and different developmental stages to investigate the transcriptome dynamics of *R2R3-MYB* genes in the allopolyploid across eight homoeologous chromosome pairs, of which 25% of the *R2R3-MYB* genes displayed allelic expression dominance in all samples. The number of expression dominant genes in the A, B, C, and D genomes was 84, 93, 82, and 79, respectively. Further, the allelic genes were compared in pairs, including A-B, A-C, A-D, B-C, B-D, and C-D (Fig. 7a). Both the number of dominant genes in a single set of homoeologous chromosomes and the pairwise comparison of alleles showed no significant allelic dominance. Captivatingly, the number of dominant genes in the stem was more than that in the leaf in each allelic pair comparison. For four sets of homoeologous chromosomes, the percentages increase was 46.5, 90.6, 10.2, 143.4%, corresponding to A, B, C, and D genomes, respectively, and the overall average rise was 64.5%. The transcriptional expression of allelic genes in the stem tissues showed significant differences among different alleles than those in the leaf tissues. Allelic expression dominant genes derived predominantly from stem transcriptomes. Selective pressure analysis demonstrated that the Ka/Ks median values of dominant allelic pairs in the stem and leaf were mainly in the range of 0.3–0.5 (Fig. 7b). The number of dominant genes in the stem with positive selection (Ka/Ks > 1) was twice that in the leaf, indicating MYB dominant genes evolved faster. MYB allelic genes with median Ka/Ks in the range from 0.4 to 0.6 were divided into three categories: dominant, subordinate, and neutral alleles, of which neutral genes were the majority (Fig. 7c). The differentially expressed dominant and subordinate genes might contribute to allelic variation that affected gene expression, function, and phenotype.

## Discussion

Gene duplication played an essential role in gene expansion and functional diversification in the genetic revolution and phenotypic evolution [40]. A total of 202 *SsR2R3-MYB* genes were identified, the second-highest number of these genes among the 21 essential plant species (displayed in Fig. 1). The number of *R2R3-MYB* in sugarcane was far more than the other members of the grass family. Nevertheless, sugarcane with octoploid nature had a higher number of *MYB* genes compared with the other species. The significant enrichment of *SsMYB* genes probably was affected by two rounds of whole-genome duplication, including allopolyploidization followed by autopolyploidization [41], or two rounds of autopolyploidization [3]. In grasses, 11 (7.09%) genes in *O. sativa* were derived from tandem duplications, 26 (21.31%) in *B. distachyon*, and 24 (15%) in *Z. mays*, while 44 (28.38%) segmental gene pairs were derived from segmental duplications in *O. sativa*, 34 (45.08%) in *B. distachyon*, and 19 (24%) in *Z. mays*, respectively [42]. The duplication of genes distribution indicated that the *MYB* genes family expansion in *S. spontaneum* could be attributed to these duplication events.

The large *R2R3-MYB* gene family resulted from duplication events, and autopolyploidization demonstrated diverse functions in plant-specific processes. Some genes specially expressed in stem tissues were concentrated in the stem prophase, indicating that these *MYB* genes might regulate biological processes related to stem development. Stem morphogenesis is tightly associated with the formation and lignification of the secondary wall (the central mechanical tissue in the stems of grass species) [43]. Indeed, some MYB transcription factors are identified to be involved in sugarcane stem development. A previous study revealed that 7 *ScMYB* genes were correlated with lignin content and biosynthesis [44]. *ShMYB78* has been recognized as an activator of suberin biosynthesis and regulates suberin deposition [45]. In *Arabidopsis*, the asymmetric leaves1 (*as*1) gene encoding an MYB protein-mediated stem cell function interacted with meristematic genes to regulate the shoot morphogenesis [46]. Furthermore, a group of rice and maize MYB genes (*OsMYB46* and *ZmMYB46*) activated the transcription of secondary cell wall biosynthesis and probably interacted with secondary wall-associated *NAC* genes [43]. The role of these *SsMYB* genes in stem development might provide potential genetic resources for sugarcane breeding.

MYB genes also play an important role in leaf development in grasses. In maize, a group of MYB was recognized to be involved in leaf development, as indicated by expression gradients. *Myb-ZmRS2*, *MYB60,* and *MYB61* influence adaxial/abaxial polarity and stomata patterning [47]. Moreover, some *ZmMYBs* are highly expressed in the transition zone, affecting secondary cell wall and lignin production [33]. The Class I containing 24 *SsR2R3-MYB* genes were also inferred to have similar functions. A few MYB genes were identified as C_4_ regulators and correlated with C_4_ photosynthetic cell type-specific gene expression. An MYB gene encoding GRMZM2G130149, which regulates the transcription of phosphoenolpyruvate carboxykinase (PEPCK) in *Z. mays*, was categorized as a C_4_ transcription factor [34]. Three putative C_4_ transcription factors (*SsMYB169*, *SsMYB181*, and *SsMYB192*) identified in this study might play a potential role in forming photosynthetic organs and regulating the C_4_ photosynthetic pathway. The *LATE ELONGATED HYPOCOTYL* (*LHY*) gene, encoding an MYB transcription factor, regulated circadian rhythms in *Arabidopsis*, and *MYB-LHY* was involved in circadian photoperiod [48]. In sugarcane, nine candidate *MYB* genes with high expression were associated with the circadian cycle and therefore performed similar functions. The genes associated with leaf development showed relatively low expression levels (FPKM< 10) than those linked with the stem tissues, hinting at a stem-related expression dominance for most *SsMYB* genes.

Drought is one of the main factors restricting sugarcane growth and sugar production [49]. Identifying particular and novel candidate genes is a great strategy to improve stress tolerance in sugarcane in this context. Certain MYB transcription factors, for instance, *MYB_2* [50], *SoMYB18* [51], *ScMYB2S1* & *2* [52], and *ScMYBAS1* [53], have been associated with the response to drought-induced stress in sugarcane. Six *MYB* genes were identified as a differential expression in this study, helping understand the sugarcane drought tolerance mechanism.

Following pathogen invasions, plants turn on a series of plant defense mechanisms. MYB transcription factors play a facilitating role in disease resistance by regulating plant hormone metabolism and mediating systemic resistance [54]. *AtMYB30* [55], *AtMYB96* [20], and *SpMYB* [56] have already been reported to be involved in disease resistance. Several defense-related MYB candidate genes were identified against pokkah boeng disease and mosaic disease of sugarcane. Hence, *MYB* genes are a component of plant defense mechanisms against fungal and viral pathogens.

Polyploids are widely distributed among plants, and about 70% of the angiosperms have experienced one or more polyploidization events during their evolution. As a plant genome evolutionary force, polyploidization plays an essential role in speciation and genomic plasticity. In this study, homologous expression dominant genes Ka/Ks of autopolyploid sugarcane were higher than those of neutral genes, consistent with the report of allopolyploid of *B. juncea* [57]. The asymmetric evolution of alleles facilitated differential expression, then affected plant biological processes. No significant differences were detected between the four unichromosomal genomes A, B, C, and D for MYB dominance. However, MYB homologous expression of dominant genes was more remarkable in number in the stem tissues than that in the leaf, and the former were subject to selection pressures with more powerful, implying that MYB stems dominant genes intensified selection in sugarcane. Not surprisingly, the transcription level of MYB genes more significantly enriched in the stem. The transcriptional advantages of these MYB homologous expression dominant genes in stem tissues might provide new insights for facilitating polyploid crop breeding for sugarcane.

## Conclusions

It is the first time deciphering the phylogeny, gene structure, and expression of the MYB family in *S. spontaneum*. Genome-wide expression analysis demonstrated that *SsMYB* genes were involved in stem development and stress response. The MYB genes might be engineered to adjust important sugarcane traits, and therefore, these genes would be a promising target for sugarcane genetic improvement.

## Materials and methods

### Obtainment of MYB genes

The autopolyploid sugarcane *S. spontaneum* L. genomic sequence was published in 2018 and available online (http://www.life.illinois.edu/ming/downloads/Spontaneum_genome/). The Hidden Markov Model (HMM) profile of the MYB DNA-binding domain (PF00249) downloaded from Pfam database (http://pfam.xfam.org/) [58] was used to search protein sequences containing MYB domain by hmm search program (HMM3.0) [59]. Then, putative MYB proteins were further screened through the NCBI-CDD database to investigate the former protein sequences and delete the proteins with incomplete domains. *SbR2R3-MYB* genes were obtained by performing the same sugarcane method without publicly available data for sorghum MYB genes. Data for sorghum protein sequences (the newest version of Sbicolor_454_v3.1.1.) were downloaded from the plant genome website Phytozome (https://phytozome.jgi.doe.gov/). Finally, we identified 418 *SsMYB* genes and 252 *SbMYB* genes (Table S1), including 202 *SsR2R3-MYB* genes and 125 *SbR2R3-MYB* genes, belonging to haplotype genes. All SsMYBs and SbMYBs sequences were placed in the Supplementary FASTA file. A plant phylogeny tree was constructed by the TimeTree Database [60]. The distribution of MYB family genes in 19 plant species were demonstrated on the previously published reports: *Ostreococcus lucimarinus*, *Volvox carteri* and *Chlamydomonas reinhardtii* from PlantTFDB (http://planttfdb.cbi.pku.edu.cn/), and a public plant transcription factor database [61], including *Physcomitrella patens* [62], *Oryza sativa* [63], *Brachypodium distachyon* [26], *Zea mays* [42], *Ananas comosus* [64], *Vitis vinifera* [65], *Arabidopsis thaliana* [63], *Brassica napus* [66], *Glycine max* [67], *Medicago truncatula* [68], *Pyrus bretschneideri* [69], *Rosa chinensis* [70], *Populus trichocarpa* [71], *Beta vulgaris* [72], *Solanum tuberosum* [73], and *Solanum lycopersicum* [74].

### Phylogenetic analysis

To generate the phylogenetic trees of MYB transcription factor family genes, multiple protein sequence alignment was performed through the MATTF program (https://mafft.cbrc.jp/alignment/server/index.html) [75] with the reported 88 rice and 138 Arabidopsis *R2R3-MYB* proteins [63], respectively. Moreover, different phylogenetic trees were constructed via the maximum likelihood method using software FastTree2 [76]. Neighbor-joining phylogenetic trees of sugarcane and sorghum were performed using MEGA7.0 [77].

### Naming *R2R3-MYB* genes and gene structure

Because of the autopolyploid nature of sugarcane (*S. spontaneum*), the identified *SsR2R3-MYB* genes partly possessed several alleles. The representative gene models for different alleles were screened by comparing the phylogenetic relationship and protein identity with sorghum homology protein and paralogs. Tandem replication genes and paralogs were regarded as new, which gene IDs were followed by P and T, respectively. The 202 representative *SsR2R3-MYB* genes were named from *SsMYB1* to *SsMYB202* according to their physical position on the chromosomes. Subsequently, allele names were supplemented with numbers (e.g., The *Sspon.01G0002470-1A* gene located at the top of chromosome 1A is *MYB1–1*, and *Sspon.01G0002470-2D* is named as *MYB1–2*). In general, *MYB1–1* as a representative gene model was directly regarded as *MYB1*. The naming method of sorghum MYB genes was also treated like that of *S. spontaneum.*

*SsR2R3-MYB* genes and CDS sequences come from the newest version of Sspon.v20190103. The domain location was derived from the previous hmm search results. Gene structures were displayed using the Gene Structure Display Server (GSDS2.0) [78], consisting of the CDS region, intron region, and MYB domain. Each gene structure was arranged according to the phylogenetic location.

### Collinearity analysis

Utilizing MCScanX analysis [79], collinearity relationships of *SsR2R3-MYB* genes and classifier program were used to sort gene duplication types. The identified collinear gene pairs were mapped to their respective locus in the *S. spontaneum* genome in a circular diagram using Circos 0.69 [80].

### Regulatory element of upstream sequences

The 2000 bp upstream sequences were extracted from *SsR2R3-MYB* genes to the PlantCARE website, plant promoter, and *cis*-element database [81]. Then, we used them to predict regulatory motifs and estimate potentially related functions.

### Abundant RNA-seq data showing gene expression

To analyze *SsR2R3-MYB* gene expression profiles thoroughly, 60 RNA-seq data were conducted to decipher their expressions from our lab and cooperative labs. Tissue and development transcriptome contained RNA-seq data of 16 samples, including leaf, stem, three different development stages viz. seeding (35-day-old), pre-maturity (9-month-old), and maturity (12-month-old) stages in *S. spontaneum* [82]. The leaf development transcriptome was derived from the second leaf alone, the ligule on 11-day-old seedlings; 15 cm leaves were selected and cut into 15 pieces with one segment per centimeter [83]. Mature leaves corresponding to ligule in *S. spontaneum*, over 12-month-old, were selected to supply circadian rhythm transcriptome using 19-time points, i.e., 2 h intervals apart from 6:00 am to the second day 4:00 am, and 4 h apart from 6:00 am to the third day 6:00 am.

RNA-seq were extracted from the drought-treatment sugarcane of FN95–1702, a new sugarcane variety for both sugar and energy, bred by Fujian Agriculture and Forestry University. Sugarcane grown to 4–5 leaves was subjected to the natural drought stress treatment in the greenhouse. The mild drought was characterized by soil relative water content of about 55% ~ 60% after 6 days, and severe drought by 25% ~ 30% after 12 days. After a severe drought, rehydration was done, and relative water content was kept around 75% ~ 85%, and then leave samples were retaken (5 days later). The *R2R3-MYB* gene expression profiles were obtained by Blast mapping to express data with transcripts of unreferenced genomes. RNA-seq for pokkah boeng disease was extracted from hybrid sugarcane ZZ1, which is highly resistant to smut disease but highly susceptible to pokkah boeng disease. According to the severity of the diseased leaves, pokkah boeng disease was divided into five grades from 0 to 5. The mildly diseased leaves (1 or 2 grades) and severely diseased leaves (4 or 5 grades) were selected for analysis, while healthy leaves were used as control (CK). Three samples were extracted for RNA-seq of sugarcane mosaic disease. For the infection experiment, sugarcane grown through virus-free tissue culture was used, and then leaves corresponding to ligule were collected 1 month after the infection, while the control plants were not infected. An expression ratio > 2 (adjusted *p*-value< 0.05) was considered statistically significant for evaluating differentially expressed genes.

### Quantitative RT-PCR

*S. spontaneum* was planted in Multifunctional Specimen Garden, Institute of Agriculture, Guangxi University. The stem-3 at the third internode and mature leaves were collected for comparing the difference of relative expression between stem and leaf. Pro-stem is short for prophase stem, in which the samples were taken from the stem precursor tissue wrapped in the leaf sheath and located on the upper part of the stem with prominent stem nodes. Combining with stem-3, stem-6, stem-9, and mature leaves was used to verify the expression during the prophase of stem formation. Total RNA was carried out using TRIZOL reagent (Takara), employing the corresponding protocol. The qualified RNA was reverse transcribed to produce cDNA using PrimeScript™ RT reagent Kit with gDNA Eraser reagent (Takara, Japan). Primers were designed by qPCR-PrimerQuest Tool, and qPCR primers were shown in Table S8. Glyceraldehyde-3-phosphate dehydrogenase gene (GAPDH) was selected as a reference gene [84]. The real-time qPCR with three biological replications was performed with SYBR green on Roche Lightcyler® 480 instrument using 2 × TB Green Mix (Takara). The reaction profile was as follows: 95 °C for 30s, followed by 40 cycles of 95 °C for 10s, 60 °C for 30s, and 95 °C for 10s. The relative expression levels were calculated by the 2^-△△^CT method.

## Supplementary Information

**Additional file 1: Table S1.** Members of identified MYB family in *S. spontaneum*.

**Additional file 2: Table S2.** The classification of the SsR2R3-MYB family by phylogenetic tree. The 15 subgroups of the SsR2R3-MYB family by phylogenetic classification with rice.

**Additional file 3: Table S3.** Members of identified MYB family in Sorghum.

**Additional file 4: Table S4.** Collinear gene pairs in the SsR2R3-MYB gene family.

**Additional file 5: Table S5.** Tandem duplication genes and segmental duplication genes in the SsR2R3-MYB gene family.

**Additional file 6: Table S6.** The expression value of SsR2R3-MYB genes in both temporal and spatial models. FPKM values of MYB genes in tissue and developmental stages, leaf developmental gradient, and circadian rhythm.

**Additional file 7: Table S7.** The expression value of DEGs of SsR2R3-MYB genes in stress response.

**Additional file 8: Table S8.** SsMYB and SbMYB sequences.

**Additional file 9: Table S9.** Gene primers of expression quantified by qPCR.

**Additional file 10: Figure S1.** Phylogenetic tree of R2R3-MYB subgroup members from *S. spontaneum* and Arabidopsis.

**Additional file 11: Figure S2.** Comparison of phylogeny and gene structure of R2R3-MYB gene between *S. spontaneum* and *S. bicolor*. (A), (B), (C), (D) continue to supplement Fig. 3a in turn.

**Additional file 12: Figure S3.** Heat map of significant DEGs with tissue specificity. Highly expressed in prophase of stem formation (A) and whole stem development period (B).

**Additional file 13: Figure S4.** Relative expression level quantified by RT-qPCR. High transcripts of MYB gene in prophase of stem formation (A) and whole stem development period (B).

**Additional file 14: Figure S5.** Sample diagram for RT-qPCR.

## Data Availability

All data generated or analyzed during this study were included in supplementary information files. Genomic data of sugarcane and sorghum for testing were obtained from the autopolyploid *S. spontaneum* L. genome (http://www.life.illinois.edu/ming/downloads/Spontaneum_genome/) and *S. bicolor* genome (https://phytozome-next.jgi.doe.gov/info/Sbicolor_v3_1_1). The domain architecture of the MYB genes was downloaded from the Pfam database (http://pfam.xfam.org/family/PF00249/hmm). The sequencing data of sugarcane pokkah boeng disease: SRP127969 (https://www.ncbi.nlm.nih.gov/sra/SRP127969); and sugarcane mosaic virus disease: SRR10058145, SRR10058144 in the GenBank database. RNA-seq of tissues and development stage, leaf segments, and circadian rhythms were downloaded from sugarcane public database (http://sugarcane.zhangjisenlab.cn/sgd/html/mRNA.html).

## References

[1] FAO F (2019). Food and Agriculture Organization of the United Nations - Statistic Division.

[2] D’Hont A, Grivet L, Feldmann P, Rao S, Berding N, Glaszmann JC (1996). Characterisation of the double genome structure of modern sugarcane cultivars (Saccharum spp.) by molecular cytogenetics. MGG. Mol Gen Genet.

[3] Saccharum L, Zhang J, Zhang X, Tang H, Zhang Q, Hua X (2018). Allele-defined genome of the autopolyploid. Nat Genet.

[4] Stracke R, Werber M, Weisshaar B (2001). The R2R3-MYB gene family in Arabidopsis thaliana. Curr Opin Plant Biol.

[5] Bilaud T, Koering CE, Binet-Brasselet E, Ancelin K, Pollice A, Gasser SM, Gilson E (1996). The Telobox, a Myb-related Telomeric DNA binding motif found in proteins from yeast, Plants and Human. Nucleic Acids Res.

[6] Kerstetter RA, Bollman K, Taylor RA, Bomblies K, Poethig RS (2001). KANADI regulates organ polarity in Arabidopsis. Nature.

[7] Yakir E, Hilman D, Kron I, Hassidim M, Melamed-Book N, Green RM (2009). Posttranslational regulation of CIRCADIAN CLOCK ASSOCIATED1 in the circadian oscillator of Arabidopsis. Plant Physiol.

[8] Ito M, Araki S, Matsunaga S, Itoh T, Nishihama R, Machida Y, Doonan JH, Watanabe A (2001). G2/M-phase-specific transcription during the plant cell cycle is mediated by c-Myb-like transcription factors. Plant Cell.

[9] Haga N, Kato K, Murase M, Araki S, Kubo M, Demura T (2007). R1R2R3-Myb proteins positively regulate cytokinesis through activation of KNOLLE transcription in *Arabidopsis thaliana*. Development.

[10] Dai X, Xu Y, Ma Q, Xu W, Wang T, Xue Y, Chong K (2007). Overexpression of an R1R2R3 MYB gene, OsMYB3R-2, increases tolerance to freezing, drought, and salt stress in transgenic Arabidopsis. Plant Physiol.

[11] Dubos C, Stracke R, Grotewold E, Weisshaar B, Martin C, Lepiniec L (2010). MYB transcription factors in Arabidopsis. Trends Plant Sci.

[12] Dias AP, Braun EL, McMullen MD, Grotewold E (2003). Recently duplicated MAIZE R2R3 MYB genes provide evidence for distinct mechanisms of evolutionary divergence after duplication. Plant Physiol.

[13] Rosinski JA, Atchley WR (1998). Molecular evolution of the Myb family of transcription factors: evidence for polyphyletic origin. J Mol Evol.

[14] Zhang P, Chopra S, Peterson T (2000). A segmental gene duplication generated differentially expressed myb-homologous genes in maize. Plant Cell.

[15] Waites R, Selvadurai HRN, Oliver IR, Hudson A (1998). The PHANTASTICA gene encodes a MYB transcription factor involved in growth and Dorsoventrality of lateral organs in Antirrhinum. Cell.

[16] Jin H, Martin C (1999). Multifunctionality and diversity within the plant MYB-gene family. Plant Mol Biol.

[17] Bender J, Fink GR (1998). A Myb homologue, ATR1, activates tryptophan gene expression in Arabidopsis. Proc Natl Acad Sci U S A.

[18] Urao T, Yamaguchi-Shinozaki K, Urao S, Shinozaki K (1993). An Arabidopsis myb homolog is induced by dehydration stress and its gene product binds to the conserved MYB recognition sequence. Plant Cell.

[19] He Q, Jones DC, Li W, Xie F, Ma J, Sun R (2016). Genome-wide identification of R2R3-MYB genes and expression analyses during abiotic stress in gossypium raimondii. Sci Rep.

[20] Seo PJ, Park C-M (2010). MYB96-mediated abscisic acid signals induce pathogen resistance response by promoting salicylic acid biosynthesis in Arabidopsis. New Phytol.

[21] McCarthy RL, Zhong R, Ye Z-H (2009). MYB83 is a direct target of SND1 and acts redundantly with MYB46 in the regulation of secondary Cell Wall biosynthesis in Arabidopsis. Plant Cell Physiol.

[22] Abe H, Urao T, Ito T, Seki M, Shinozaki K, Yamaguchi-Shinozaki K (2003). Arabidopsis AtMYC2 (bHLH) and AtMYB2 (MYB) function as transcriptional activators in abscisic acid signaling. Plant Cell.

[23] Seo PJ, Lee SB, Suh MC, Park M-J, Go YS, Park C-M (2011). The MYB96 transcription factor regulates cuticular wax biosynthesis under drought conditions in Arabidopsis. Plant Cell.

[24] Bowring SA, Grotzinger JP, Isachsen CE, Knoll AH, Pelechaty SM, Kolosov P (1993). Calibrating rates of early Cambrian evolution. Science.

[25] McInerney FA, Wing SL (2011). The Paleocene-Eocene thermal maximum: a perturbation of carbon cycle, climate, and biosphere with implications for the future. Annu Rev Earth Planet Sci.

[26] Chen S, Niu X, Guan Y, Li H (2017). Genome-wide analysis and expression profiles of the MYB genes in Brachypodium distachyon. Plant Cell Physiol.

[27] Paterson AH, Bowers JE, Bruggmann R, Dubchak I, Grimwood J, Gundlach H, Haberer G, Hellsten U, Mitros T, Poliakov A, Schmutz J, Spannagl M, Tang H, Wang X, Wicker T, Bharti AK, Chapman J, Feltus FA, Gowik U, Grigoriev IV, Lyons E, Maher CA, Martis M, Narechania A, Otillar RP, Penning BW, Salamov AA, Wang Y, Zhang L, Carpita NC, Freeling M, Gingle AR, Hash CT, Keller B, Klein P, Kresovich S, McCann MC, Ming R, Peterson DG, Mehboob-ur-Rahman, Ware D, Westhoff P, Mayer KFX, Messing J, Rokhsar DS (2009). The Sorghum bicolor genome and the diversification of grasses. Nature.

[28] McCormick RF, Truong SK, Sreedasyam A, Jenkins J, Shu S, Sims D (2018). The Sorghum bicolor reference genome: improved assembly and annotations, a transcriptome atlas, and signatures of genome organization. Plant J.

[29] Kieffer M, Stern Y, Cook H, Clerici E, Maulbetsch C, Laux T, Davies B (2006). Analysis of the transcription factor WUSCHEL and its functional homologue in Antirrhinum reveals a potential mechanism for their roles in meristem maintenance. Plant Cell.

[30] Sharma P, Lin T, Grandellis C, Yu M, Hannapel DJ (2014). The BEL1-like family of transcription factors in potato. J Exp Bot.

[31] Yanagisawa S, Sheen J (1998). Involvement of maize Dof zinc finger proteins in tissue-specific and light-regulated gene expression. Plant Cell.

[32] Rabinowicz PD, Braun EL, Wolfe AD, Bowen B, Grotewold E (1999). Maize R2R3 Myb genes: sequence analysis reveals amplification in the higher plants. Genetics.

[33] Li P, Ponnala L, Gandotra N, Wang L, Si Y, Tausta SL, Kebrom TH, Provart N, Patel R, Myers CR, Reidel EJ, Turgeon R, Liu P, Sun Q, Nelson T, Brutnell TP (2010). The developmental dynamics of the maize leaf transcriptome. Nat Genet.

[34] Studer AJ, Schnable JC, Weissmann S, Kolbe AR, McKain MR, Shao Y (2016). The draft genome of the C3 panicoid grass species Dichanthelium oligosanthes. Genome Biol.

[35] Wang L, Czedik-Eysenberg A, Mertz RA, Si Y, Tohge T, Nunes-Nesi A, Arrivault S, Dedow LK, Bryant DW, Zhou W, Xu J, Weissmann S, Studer A, Li P, Zhang C, LaRue T, Shao Y, Ding Z, Sun Q, Patel RV, Turgeon R, Zhu X, Provart NJ, Mockler TC, Fernie AR, Stitt M, Liu P, Brutnell TP (2014). Comparative analyses of C_4_ and C_3_ photosynthesis in developing leaves of maize and rice. Nat Biotechnol.

[36] Rott R, Bailey RA (2000). JC Comstock BC and AS. A guide to sugarcane diseases.

[37] Singh A, Chauhan SS, Singh A, Singh SB (2006). Deterioration in sugarcane due to pokkah boeng disease. Sugar Tech.

[38] Xu D-L, Park J-W, Mirkov TE, Zhou G-H (2008). Viruses causing mosaic disease in sugarcane and their genetic diversity in southern China. Arch Virol.

[39] Chauhan RP, Rajakaruna P, Verchot J (2015). Complete genome sequence of nine isolates of canna yellow streak virus reveals its relationship to the sugarcane mosaic virus (SCMV) subgroup of potyviruses. Arch Virol.

[40] Flagel LE, Wendel JF (2009). Gene duplication and evolutionary novelty in plants. New Phytol.

[41] Kim C, Wang X, Lee T-H, Jakob K, Lee G-J, Paterson AH (2014). Comparative analysis of Miscanthus and Saccharum reveals a shared whole-genome duplication but different evolutionary fates. Plant Cell.

[42] Du H, Feng B-R, Yang S-S, Huang Y-B, Tang Y-X (2012). The R2R3-MYB transcription factor gene family in maize. PLoS One.

[43] Zhong R, Lee C, McCarthy RL, Reeves CK, Jones EG, Ye Z-H (2011). Transcriptional activation of secondary wall biosynthesis by rice and maize NAC and MYB transcription factors. Plant Cell Physiol.

[44] dos Santos Brito M. Unraveling two ways of lignin biosynthesis in sugarcane culm: an overview of transcriptional regulation. Plant Biology Dept. 2012:P0738 Brazil: Ribeirao Preto, IB-UNICAMP.

[45] Figueiredo R, Portilla Llerena JP, Kiyota E, Ferreira SS, Cardeli BR, de Souza SCR, dos Santos Brito M, Sodek L, Cesarino I, Mazzafera P (2020). The sugarcane ShMYB78 transcription factor activates suberin biosynthesis in Nicotiana benthamiana. Plant Mol Biol.

[46] Byrne ME, Barley R, Curtis M, Arroyo JM, Dunham M, Hudson A, Martienssen RA (2000). Asymmetric leaves1 mediates leaf patterning and stem cell function in Arabidopsis. Nature.

[47] Husbands AY, Chitwood DH, Plavskin Y, Timmermans MCP (2009). Signals and prepatterns: new insights into organ polarity in plants. Genes Dev.

[48] Carré IA, Kim J-Y (2002). MYB transcription factors in the Arabidopsis circadian clock. J Exp Bot.

[49] Prabu G, Kawar PG, Pagariya MC, Prasad DT (2011). Identification of water deficit stress Upregulated genes in sugarcane. Plant Mol Biol Report.

[50] Souza SC. Characterization of transcription factors differentially expressed under drought conditions in sugarcane (Saccharum spp). Plant Animal Genome. 2015.

[51] Shingote PR, Kawar PG, Pagariya MC, Kuhikar RS, Thorat AS, Babu KH (2015). SoMYB18, a sugarcane MYB transcription factor improves salt and dehydration tolerance in tobacco. Acta Physiol Plant.

[52] Guo J, Ling H, Ma J, Chen Y, Su Y, Lin Q, Gao S, Wang H, Que Y, Xu L (2017). A sugarcane R2R3-MYB transcription factor gene is alternatively spliced during drought stress. Sci Rep.

[53] Fávero Peixoto-Junior R, Mara de Andrade L, dos Santos Brito M, Macedo Nobile P, Palma Boer Martins A, Domingues Carlin S (2018). Overexpression of ScMYBAS1 alternative splicing transcripts differentially impacts biomass accumulation and drought tolerance in rice transgenic plants. PLoS One.

[54] Raffaele S, Rivas S, Roby D (2006). An essential role for salicylic acid in AtMYB30-mediated control of the hypersensitive cell death program in Arabidopsis. FEBS Lett.

[55] Vailleau F, Daniel X, Tronchet M, Montillet J-L, Triantaphylidès C, Roby D (2002). A R2R3-MYB gene, AtMYB30, acts as a positive regulator of the hypersensitive cell death program in plants in response to pathogen attack. Proc Natl Acad Sci U S A.

[56] Liu Z, Luan Y, Li J, Yin Y (2016). Expression of a tomato MYB gene in transgenic tobacco increases resistance to Fusarium oxysporum and Botrytis cinerea. Eur J Plant Pathol.

[57] Yang J, Liu D, Wang X, Ji C, Cheng F, Liu B, Hu Z, Chen S, Pental D, Ju Y, Yao P, Li X, Xie K, Zhang J, Wang J, Liu F, Ma W, Shopan J, Zheng H, Mackenzie SA, Zhang M (2016). The genome sequence of allopolyploid Brassica juncea and analysis of differential homoeolog gene expression influencing selection. Nat Genet.

[58] Finn RD, Coggill P, Eberhardt RY, Eddy SR, Mistry J, Mitchell AL, Potter SC, Punta M, Qureshi M, Sangrador-Vegas A, Salazar GA, Tate J, Bateman A (2016). The Pfam protein families database: towards a more sustainable future. Nucleic Acids Res.

[59] Wheeler TJ, Eddy SR (2013). Nhmmer: DNA homology search with profile HMMs. Bioinformatics.

[60] Hedges SB, Dudley J, Kumar S (2006). TimeTree: a public knowledge-base of divergence times among organisms. Bioinformatics.

[61] Guo A-Y, Chen X, Gao G, Zhang H, Zhu Q-H, Liu X-C, Zhong YF, Gu X, He K, Luo J (2008). PlantTFDB: a comprehensive plant transcription factor database. Nucleic Acids Res.

[62] Pu X, Yang L, Liu L, Dong X, Chen S, Chen Z, et al. Genome-wide analysis of the MYB transcription factor superfamily in Physcomitrella patens. Int J Mol Sci. 2020;21(3). 10.3390/ijms21030975. 10.3390/ijms21030975 PMC7037163 32024128

[63] Katiyar A, Smita S, Lenka SK, Rajwanshi R, Chinnusamy V, Bansal KC (2012). Genome-wide classification and expression analysis of MYB transcription factor families in rice and Arabidopsis. BMC Genomics.

[64] Liu C, Xie T, Chen C, Luan A, Long J, Li C (2017). Genome-wide organization and expression profiling of the R2R3-MYB transcription factor family in pineapple (Ananas comosus). BMC Genomics.

[65] Matus JT, Aquea F, Arce-Johnson P (2008). Analysis of the grape MYB R2R3 subfamily reveals expanded wine quality-related clades and conserved gene structure organization across Vitis and Arabidopsis genomes. BMC Plant Biol.

[66] Chen B, Niu F, Liu W-Z, Yang B, Zhang J, Ma J, Cheng H, Han F, Jiang YQ (2016). Identification, cloning and characterization of R2R3-MYB gene family in canola (Brassica napus L.) identify a novel member modulating ROS accumulation and hypersensitive-like cell death. DNA Res.

[67] Du H, Yang S-S, Liang Z, Feng B-R, Liu L, Huang Y-B (2012). Genome-wide analysis of the MYB transcription factor superfamily in soybean. BMC Plant Biol.

[68] ZHENG X, YI D, SHAO L, LI C. (2017). In silico genome-wide identification, phylogeny and expression analysis of the R2R3-MYB gene family in Medicago truncatula. J Integr Agric.

[69] Feng S, Xu Y, Yang L, Sun S, Wang D, Chen X (2015). Genome-wide identification and characterization of R2R3-MYB transcription factors in pear. Sci Hortic (Amsterdam).

[70] Han Y, Yu J, Zhao T, Cheng T, Wang J, Yang W (2019). Dissecting the Genome-Wide Evolution and Function of R2R3-MYB Transcription Factor Family in *Rosa chinensis*. Genes.

[71] Wilkins O, Nahal H, Foong J, Provart NJ, Campbell MM (2009). Expansion and diversification of the Populus R2R3-MYB Family of Transcription Factors. Plant Physiol.

[72] Stracke R, Holtgräwe D, Schneider J, Pucker B, Rosleff Sörensen T, Weisshaar B (2014). Genome-wide identification and characterisation of R2R3-MYB genes in sugar beet (Beta vulgaris). BMC Plant Biol.

[73] Sun W, Ma Z, Chen H, Liu M. MYB gene family in potato (Solanum tuberosum L.): genome-wide identification of hormone-responsive reveals their potential functions in growth and development. Int J Mol Sci. 2019;20(19). 10.3390/ijms20194847. 10.3390/ijms20194847 PMC6801432 31569557

[74] Zhao P, Li Q, Li J, Wang L, Ren Z (2014). Genome-wide identification and characterization of R2R3MYB family in Solanum lycopersicum. Mol Gen Genomics.

[75] Katoh K, Standley DM (2013). MAFFT multiple sequence alignment software version 7: improvements in performance and usability. Mol Biol Evol.

[76] Price MN, Dehal PS, Arkin AP (2010). FastTree 2 – approximately maximum-likelihood trees for large alignments. PLoS One.

[77] Kumar S, Stecher G, Tamura K (2016). MEGA7: molecular evolutionary genetics analysis version 7.0 for bigger datasets. Mol Biol Evol.

[78] Hu B, Jin J, Guo A-Y, Zhang H, Luo J, Gao G (2015). GSDS 2.0: an upgraded gene feature visualization server. Bioinformatics.

[79] Thompson JD, Gibson TJ, Higgins DG (2003). Multiple sequence alignment using ClustalW and ClustalX. Curr Protoc Bioinform.

[80] Krzywinski M, Schein J, Birol I, Connors J, Gascoyne R, Horsman D, Jones SJ, Marra MA (2009). Circos: an information aesthetic for comparative genomics. Genome Res.

[81] Lescot M, Déhais P, Thijs G, Marchal K, Moreau Y, Van de Peer Y (2002). PlantCARE, a database of plant cis-acting regulatory elements and a portal to tools for in silico analysis of promoter sequences. Nucleic Acids Res.

[82] Chen Y, Zhang Q, Hu W, Zhang X, Wang L, Hua X, Yu Q, Ming R, Zhang J (2017). Evolution and expression of the fructokinase gene family in Saccharum. BMC Genomics.

[83] Li Z, Hua X, Zhong W, Yuan Y, Wang Y, Wang Z, Ming R, Zhang J (2020). Genome-wide identification and expression profile analysis of WRKY family genes in the autopolyploid Saccharum spontaneum. Plant Cell Physiol.

[84] Ling H, Wu Q, Guo J, Xu L, Que Y (2014). Comprehensive selection of reference genes for gene expression normalization in sugarcane by real time quantitative rt-PCR. PLoS One.

