# Supplementary figures and images for: Genome-wide analysis of R2R3-MYB transcription factors family in the autopolyploid Saccharum spontaneum: an exploration of dominance expression and stress response
Source: BMC Genomics. 2021 Aug 18;22:622. doi: 10.1186/s12864-021-07689-w (PMC8371785; doi:10.1186/s12864-021-07689-w)

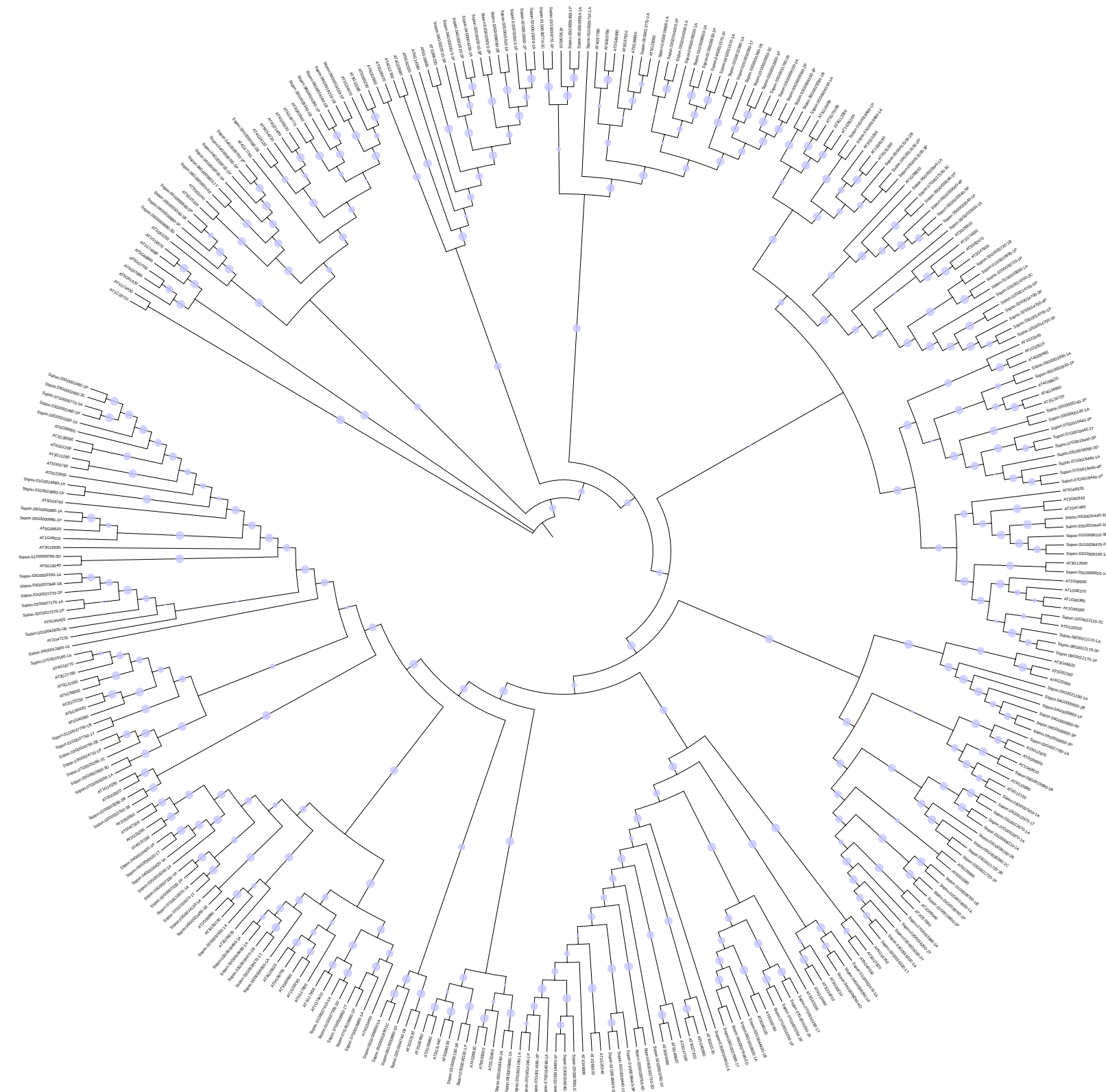

Supplement: Supplementary file 10 — Additional file 10: Figure S1. Phylogenetic tree of R2R3-MYB subgroup members from S. spontaneum and Arabidopsis. [file 12864_2021_7689_MOESM10_ESM.pdf]

A

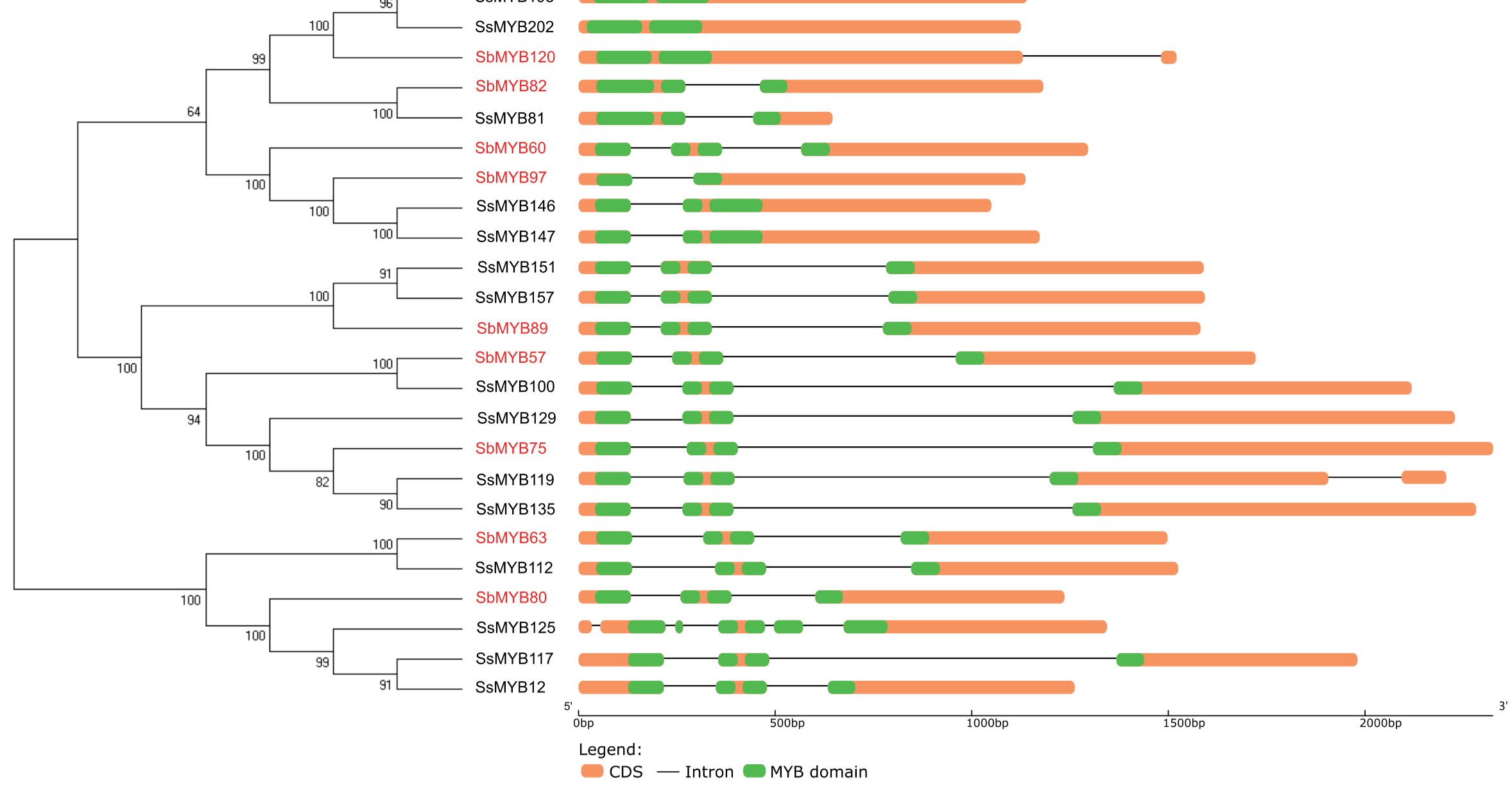

B

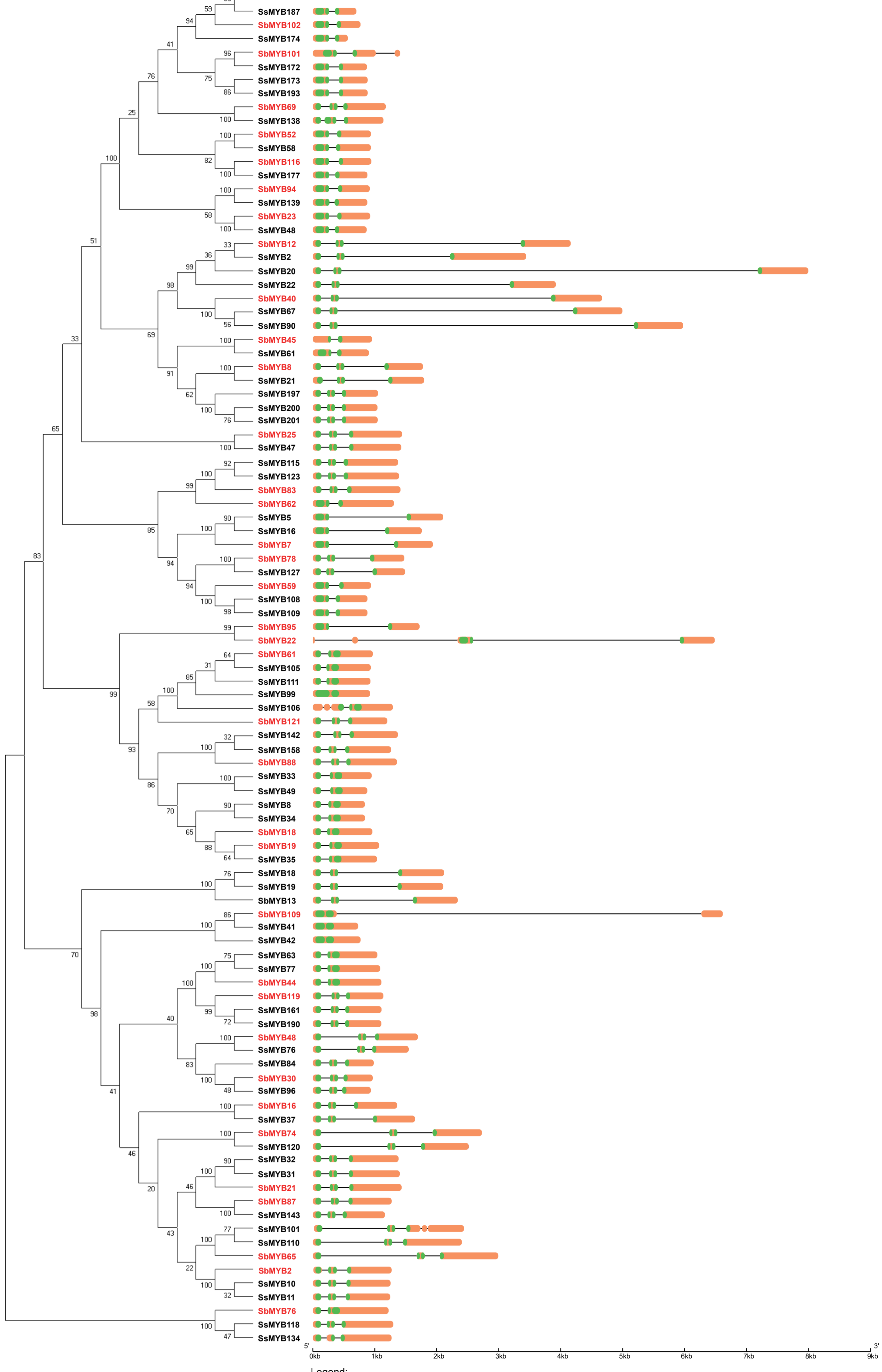

C

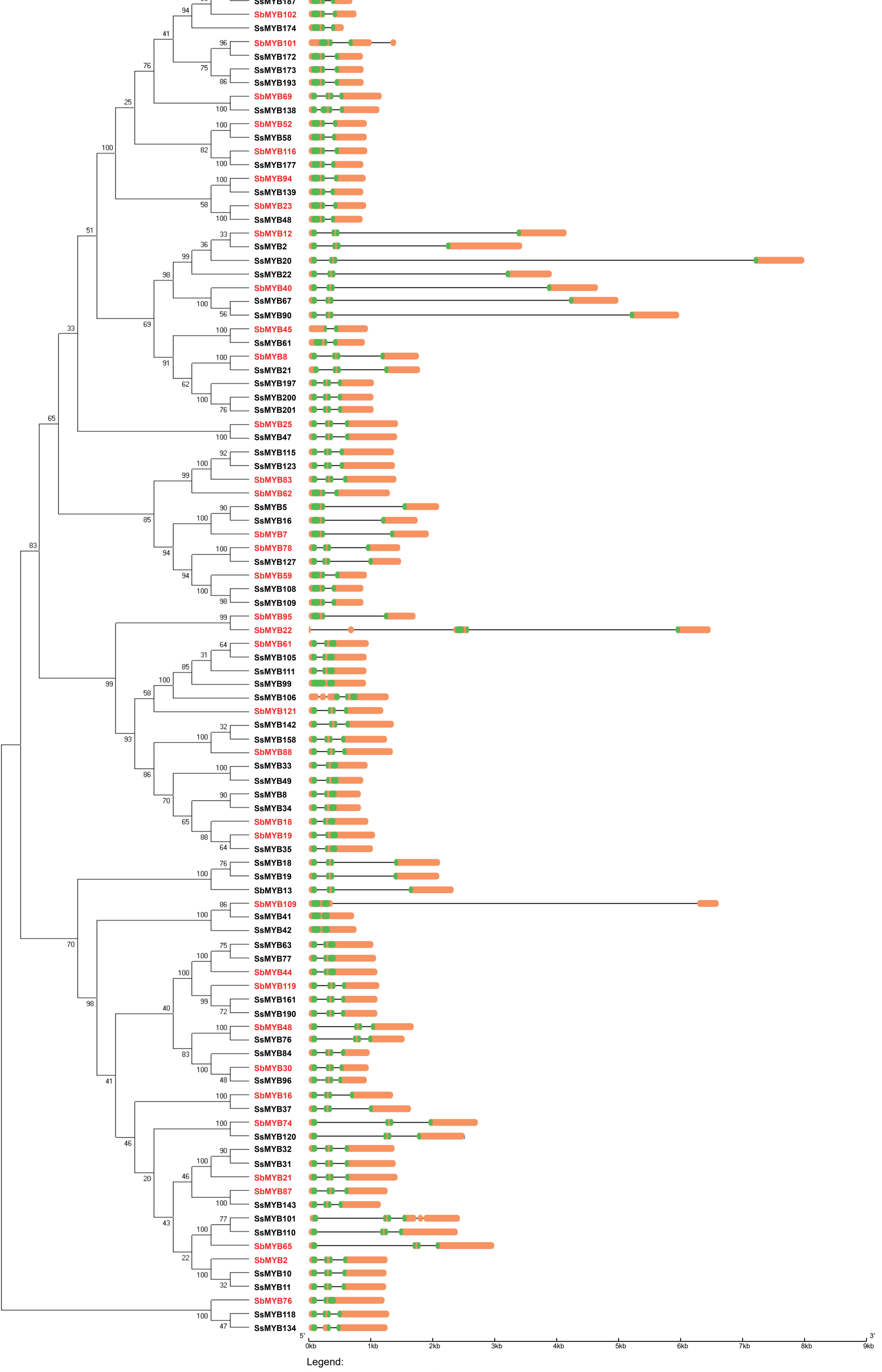

D

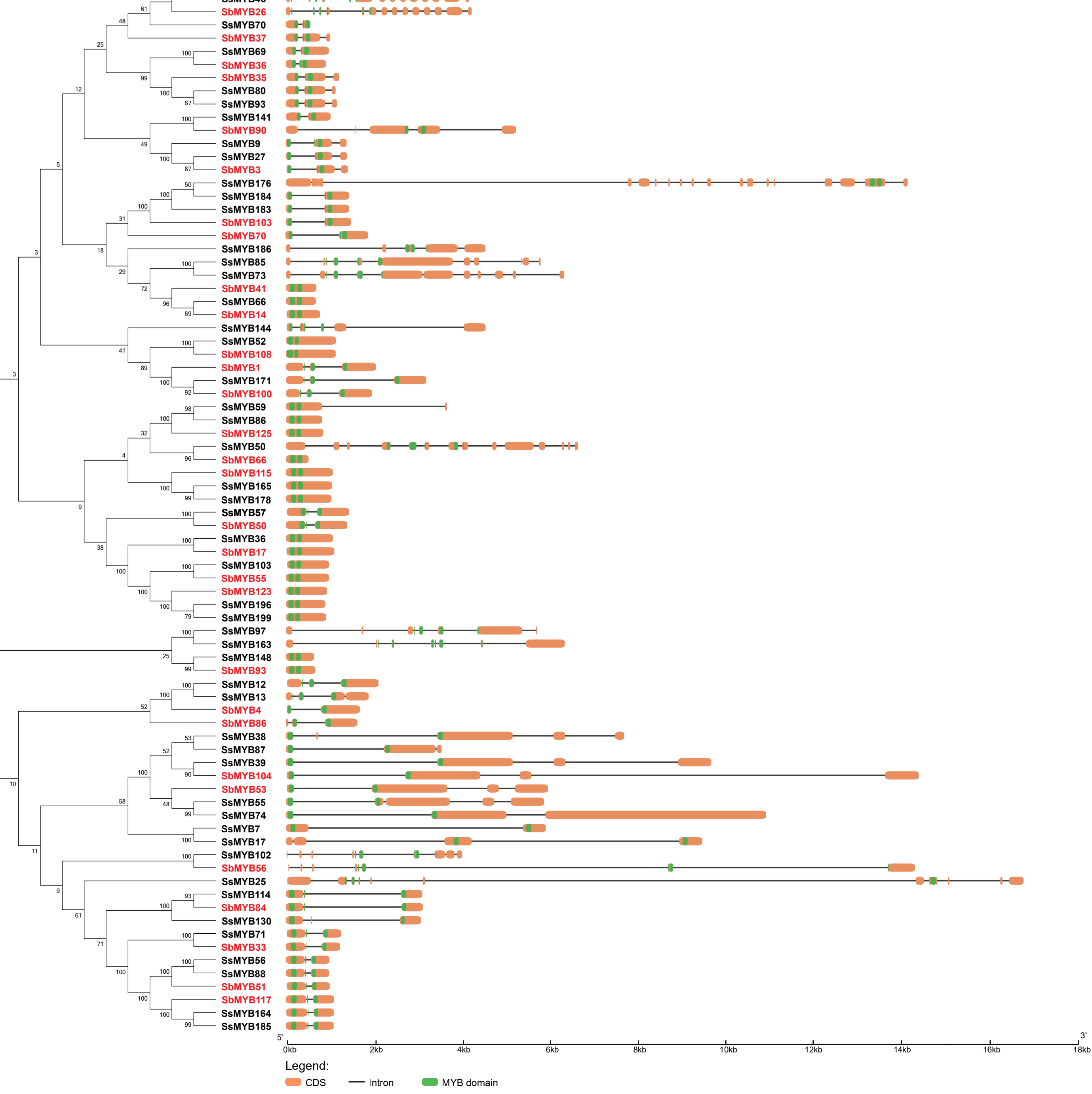

Supplement: Supplementary file 11 — Additional file 11: Figure S2. Comparison of phylogeny and gene structure of R2R3-MYB gene between S. spontaneum and S. bicolor. (A), (B), (C), (D) continue to supplement Fig. 3a in turn. [file 12864_2021_7689_MOESM11_ESM.pdf]

A

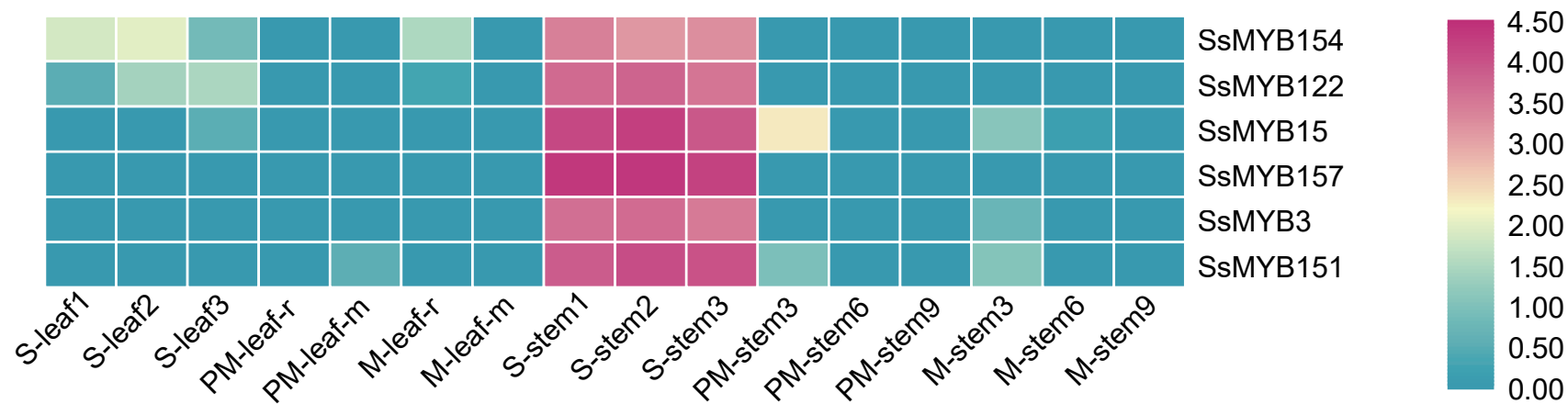

B

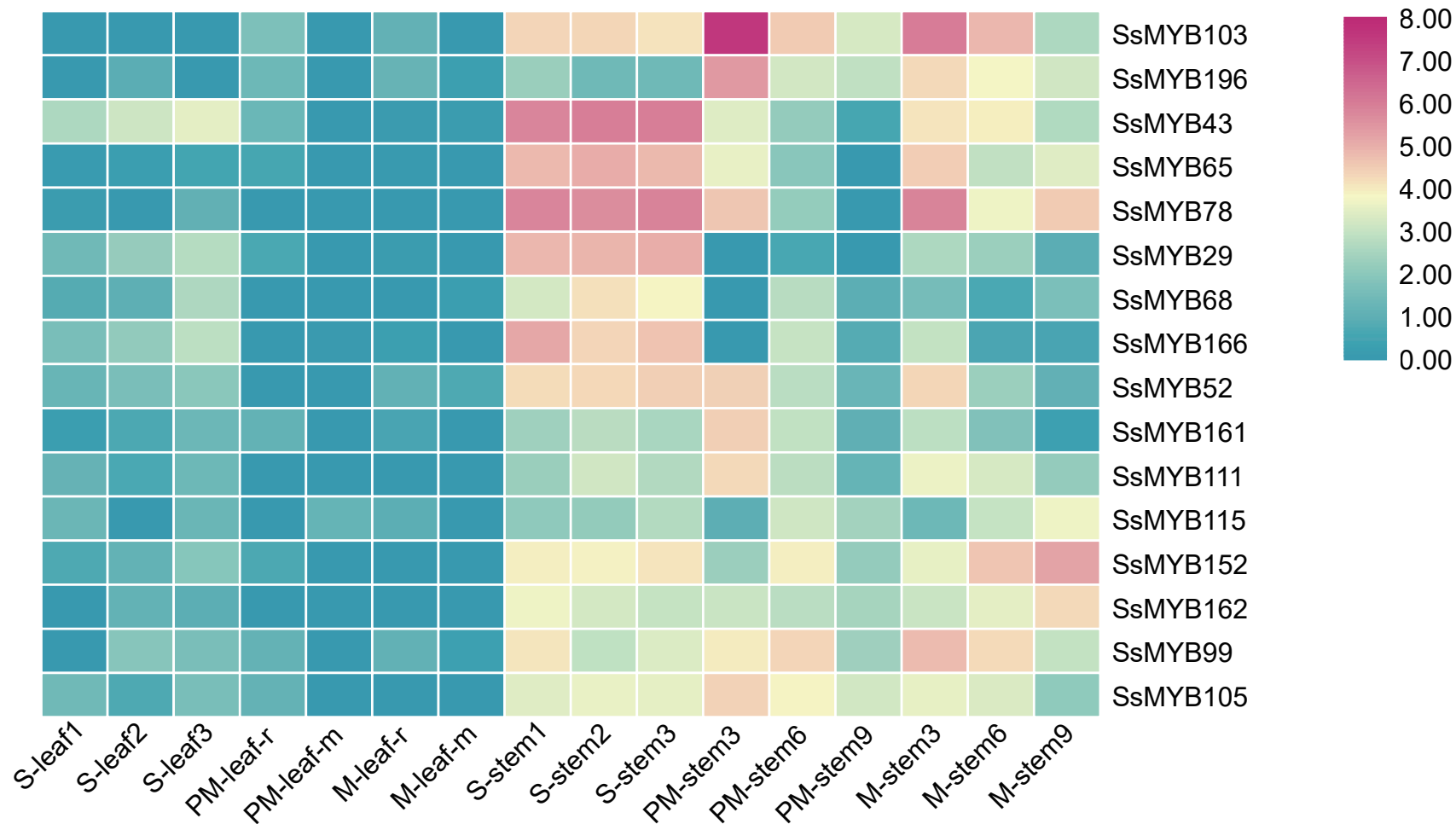

Supplement: Supplementary file 12 — Additional file 12: Figure S3. Heat map of significant DEGs with tissue specificity. Highly expressed in prophase of stem formation (A) and whole stem development period (B). [file 12864_2021_7689_MOESM12_ESM.pdf]

A

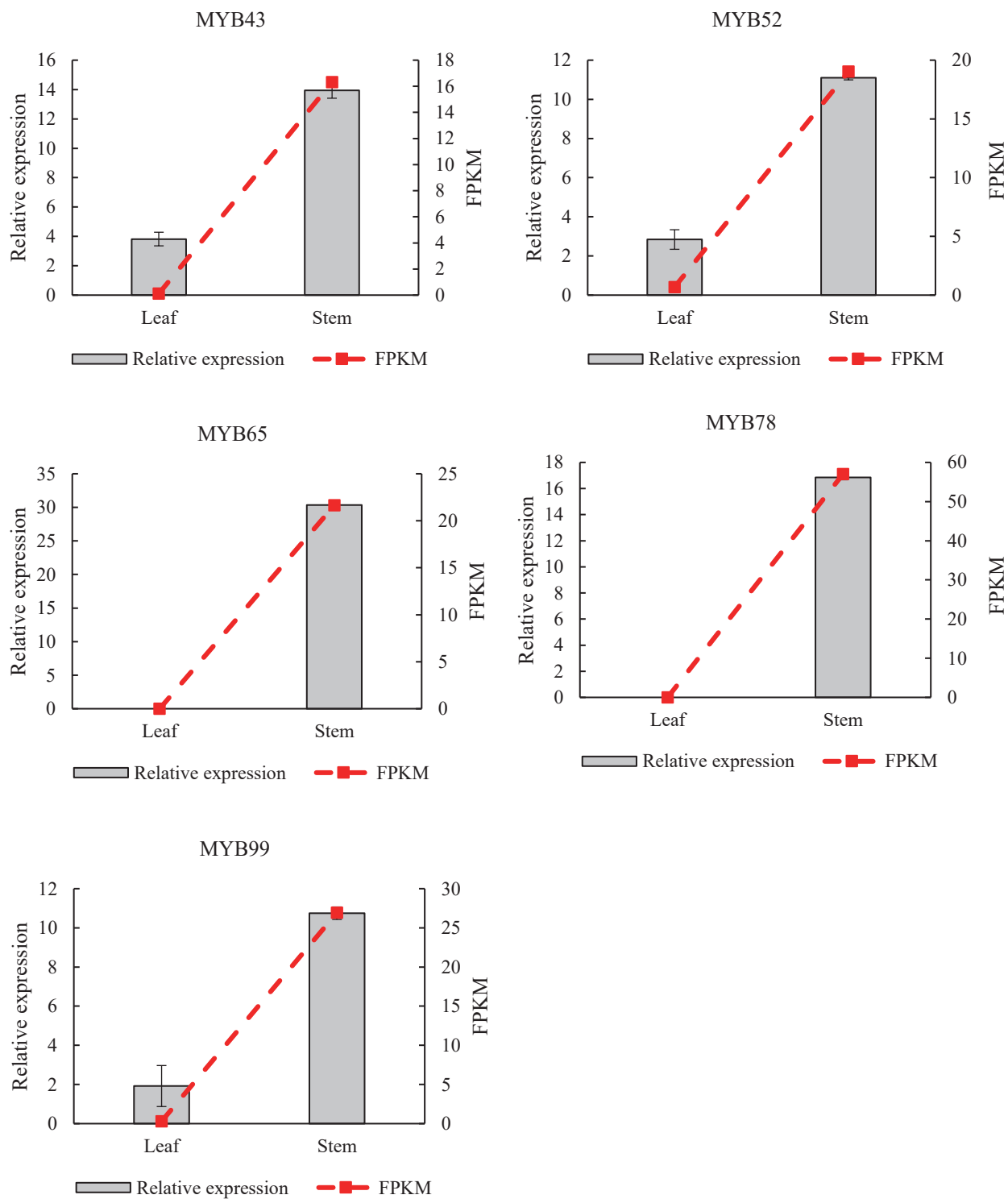

B

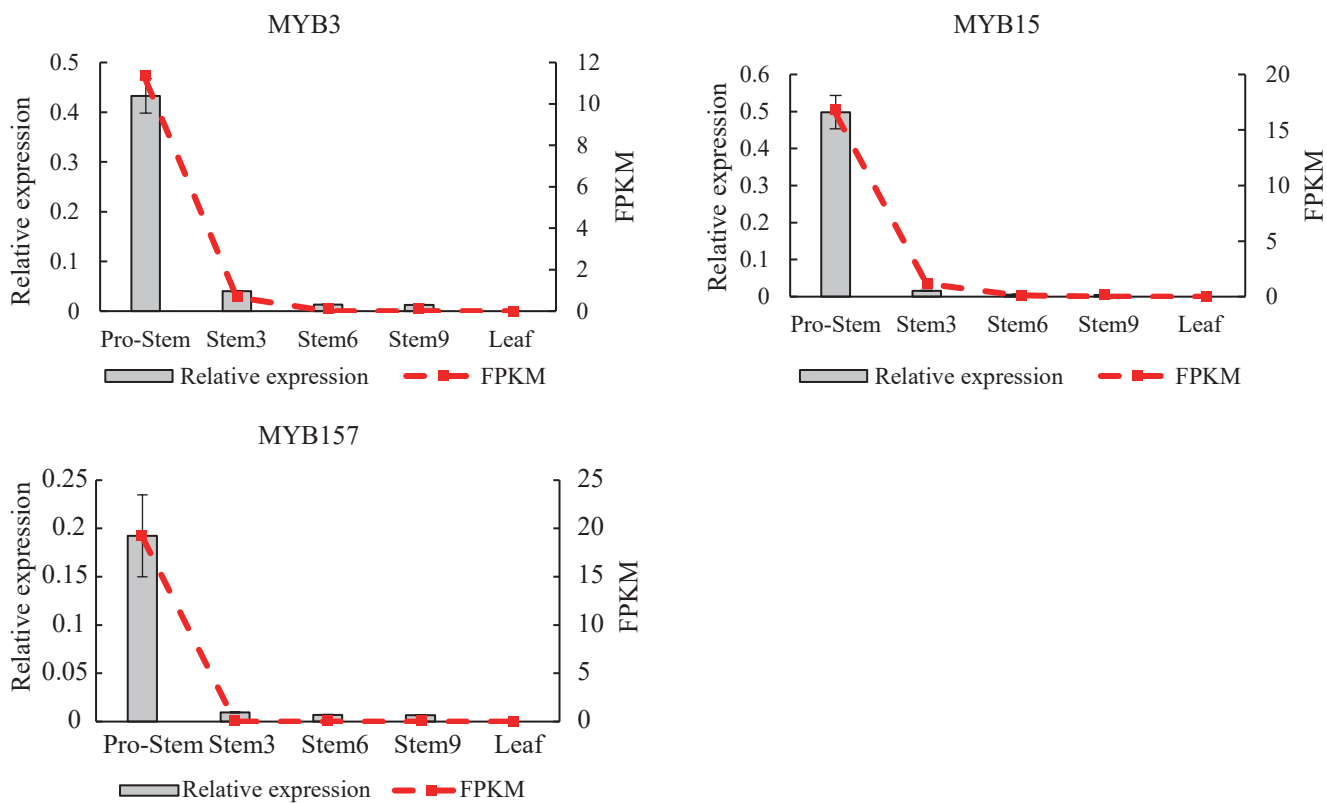

Supplement: Supplementary file 13 — Additional file 13: Figure S4. Relative expression level quantified by RT-qPCR. High transcripts of MYB gene in prophase of stem formation (A) and whole stem development period (B). [file 12864_2021_7689_MOESM13_ESM.pdf]

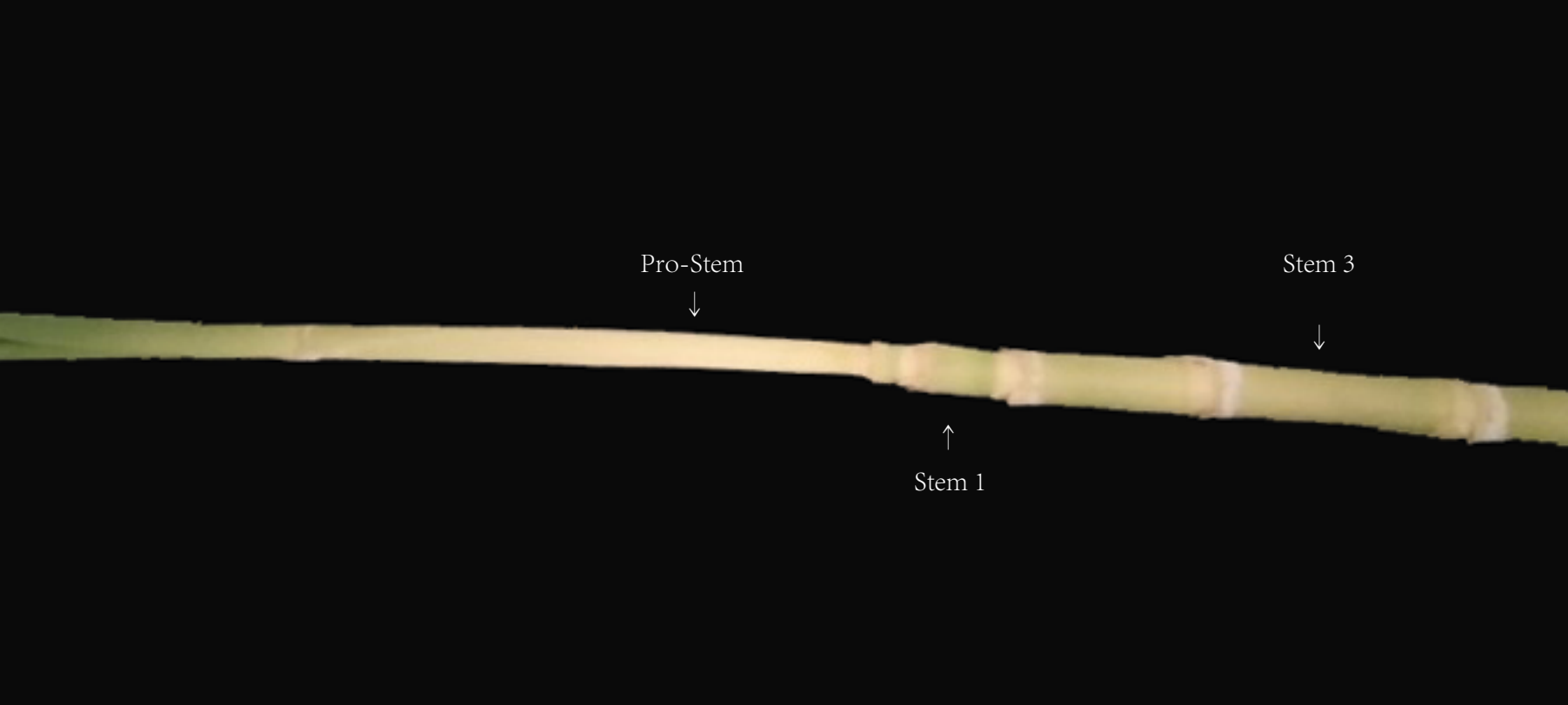

Pro-Stem

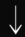

Stem 3

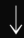

Stem 1

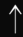

Supplement: Supplementary file 14 — Additional file 14: Figure S5. Sample diagram for RT-qPCR. [file 12864_2021_7689_MOESM14_ESM.pdf]
